# SRNR: Deep learning-based MRI super-resolution using noisy high-resolution reference data

**DOI:** 10.1162/IMAG.a.1433

**Published:** 2026-11-03

**Authors:** Muye Zhang, Hongjia Yang, Zihan Li, Jiaxin Xiao, Wanqing Liu, Yuhang He, Ting Yin, Jialan Zheng, Mingxuan Liu, Wei Liu, Congyu Liao, Berkin Bilgic, Jonathan R. Polimeni, Susie Y. Huang, Yi Liao, Ziyu Li, Haibo Qu, Qiyuan Tian

**Affiliations:** School of Biomedical Engineering, Tsinghua University, Beijing, China; Department of Radiology, West China Second University Hospital, Sichuan University, Chengdu, China; MR Research Collaborations, Siemens Healthineers Ltd., Chengdu, China; Siemens Healthineers AG, Erlangen, Germany; Department of Radiology and Biomedical Imaging, University of California San Francisco, San Francisco, CA, United States; Athinoula A. Martinos Center for Biomedical Imaging, Department of Radiology, Massachusetts General Hospital, Charlestown, MA, United States; Department of Radiology, Harvard Medical School, Boston, MA, United States; Harvard-MIT Program in Health Sciences and Technology, Massachusetts Institute of Technology, Cambridge, MA, United States; Oxford Centre for Integrative Neuroimaging, FMRIB, Nuffield Department of Clinical Neurosciences, University of Oxford, Oxford, United Kingdom

**Keywords:** convolutional neural network, denoising, Noise2Noise, pediatric neuroimaging, tissue segmentation, cortical surface reconstruction

## Abstract

Deep learning-based super-resolution (SR) techniques offer a powerful tool to enhance the spatial resolution of magnetic resonance imaging (MRI) without increasing scan time. However, most supervised SR methods rely on high-resolution, high-signal-to-noise ratio (SNR) reference data for training, which are difficult to acquire in many clinical settings, such as in time-constrained pediatric imaging. Inspired by the Noise2Noise principle, we propose SRNR, a new training paradigm that enables supervised SR using noisy high-resolution images as references. SRNR was systematically evaluated on both pediatric and adult simulated data across different noise levels of the reference data. SRNR generated super-resolved image volumes with high similarity to the high-resolution references (PSNR: 32.5–35.2 dB) and improved downstream image analysis performance, including tissue segmentation (white matter Dice coefficient: 0.951–0.960) and cortical surface reconstruction (cortical thickness absolute difference: 0.200–0.255 mm). Furthermore, SRNR was shown to be effective on clinically acquired pediatric brain MRI data, achieving improvements consistent with those observed in the simulated experiments. These results demonstrate that SRNR substantially reduces reliance on clean, high-SNR training data and enables effective SR under clinically realistic acquisition constraints, thereby improving the feasibility of deploying deep learning-based SR in pediatric MRI and related applications.

## 1 Introduction

High-resolution magnetic resonance imaging (MRI) is a cornerstone of precision medicine. It enables the detailed visualization of fine anatomical structures and the accurate, noninvasive quantification of morphological features. In clinical practice, especially in pediatric neurological disorders such as intractable epilepsy (Goyal et al., 2004; Vecchiato et al., 2025), pineal cysts (Whitehead et al., 2013), and autism (Andrews et al., 2025), high-resolution MRI can reveal subtle structural abnormalities that are often undetectable at conventional spatial resolutions or with other imaging modalities such as ultrasonography and computed tomography, thereby directly informing clinical diagnosis and treatment planning.

However, acquiring high-resolution MRI typically requires prolonged scan times to sample a larger extent of k-space. This challenge is especially pronounced in pediatric imaging, where the unfamiliar environment, confined space, and acoustic noise make it difficult for children to remain still during acquisition. As a result, sedation is frequently used to mitigate motion artifacts but is associated with substantial risks, with reported adverse event rates exceeding 10%, including respiratory complications, hypoxia, and prolonged drowsiness (S.-Z. Dong et al., 2019; Malviya et al., 2000), as well as potential long-term neurocognitive effects (Ahmad et al., 2018; Barnea-Goraly et al., 2014). Although various strategies, such as child-friendly imaging workflows (Gallo-Bernal et al., 2023; Runge et al., 2025), in-bore visual distraction systems, and sleep manipulation, have been proposed to reduce reliance on sedation, they often fail to completely eliminate the need for sedative use (Molina-Maza et al., 2022). This trade-off substantially constrains the feasibility of acquiring high-resolution image volumes in routine clinical practice and underscores the need for methods that enable high-resolution MRI without lengthy acquisitions.

Single-stack super-resolution (SR) offers an effective means of achieving high-resolution imaging without extending scan time (Li et al., 2025; Plenge et al., 2012). By reducing patient burden while maintaining or improving image quality (De Leeuw Den Bouter et al., 2022; Lyu et al., 2018; Wu et al., 2023), SR techniques have been widely adopted in biomedical imaging applications (Chaudhari et al., 2018, 2020; Du et al., 2020; Morell-Ortega et al., 2025; Ordinola et al., 2025; Ratcliffe et al., 2025; Wang et al., 2019). In recent years, deep learning-based SR methods have consistently outperformed conventional approaches (C. Dong et al., 2016; Kim et al., 2016). In particular, three-dimensional (3D) SR models exploit inter-slice spatial context to more accurately reconstruct complex anatomical structures, thereby enabling superior performance in downstream tasks such as tissue segmentation, cortical surface reconstruction, and functional connectivity analysis (Iglesias et al., 2023; Li et al., 2023; Tian, Bilgic, et al., 2021; Yang et al., 2025).

Despite these advances, the majority of supervised SR methods rely on high-resolution, high-signal-to-noise ratio (SNR) reference image volumes for training, which are difficult to acquire under routine clinical conditions. Higher spatial resolution is intrinsically incompatible with high SNR, as SNR decreases with decreasing voxel size. A common strategy to compensate for SNR degradation in high-resolution scans is to average multiple repetitions, which substantially prolongs acquisition time. For example, achieving sufficient SNR to resolve cortical anatomical structures for anatomically accurate and reliable cortical surface reconstruction from 0.6-mm isotropic T1-weighted (T1w) image volumes at 3T requires averaging 5–6 repeated scans (Zaretskaya & Polimeni, 2018). Such extended acquisitions increase vulnerability to subject motion, while subsequent co-registration and averaging can introduce additional blurring in the reference image volumes. Taken together, these constraints severely limit the scalability and clinical applicability of conventional supervised SR methods that rely on high-SNR reference data.

Recent advances in deep learning have demonstrated that high-SNR reference images traditionally required for image denoising can be replaced by noisy images during training while still achieving competitive performance. A notable example is the Noise2Noise (Lehtinen et al., 2018) framework, which shows that denoising neural networks can be trained using noisy targets, provided that the target noise is zero mean and statistically independent of the input. Under an L2 loss, the network output converges to the expectation of the target noise (i.e., zero), thereby implicitly recovering the underlying noise-free image.

In this study, we extend this concept to image volume SR and propose SRNR, a training paradigm designed to generate high-resolution, high-SNR image volumes from low-resolution MRI acquisitions using only low-quality, noisy reference targets for training. Using noise-free digital phantoms, we first conducted simulation experiments in which controlled levels of Gaussian noise were added to the training targets to systematically assess the robustness of SRNR. We then evaluated SRNR on clinically acquired pediatric brain MRI data using accelerated acquisition protocols. In addition, we investigated a practical transfer learning strategy in which SRNR was pretrained on publicly available datasets and subsequently fine-tuned using only a small number of empirical scans. Finally, we examined performance on downstream tasks, including cortical surface reconstruction and tissue segmentation.

Across all experiments, SRNR consistently generated high-quality super-resolved image volumes and faithfully recovered fine anatomical structures. The performance of models trained using noisy training targets was comparable with that of models trained with high-SNR reference data. Training data requirements can be further alleviated through transfer learning. By substantially reducing reliance on high-quality, high-SNR reference data, SRNR enables effective training directly from noisy acquisitions without the need for repeated scans. Taken together, these results establish SRNR as a practical, data-efficient solution for clinical SR MRI, enhancing both its feasibility and translational potential in routine imaging workflows.

## 2 Methods

### 2.1 SRNR training paradigm

In MRI, raw k-space measurements are corrupted by complex Gaussian noise, and the reconstructed magnitude image volumes follow a Rician distribution. Although Rician noise exhibits a non-zero mean in low-SNR regions, within the brain parenchyma where the SNR is sufficiently high, the Rician distribution can be well approximated by additive zero-mean Gaussian noise (Gudbjartsson & Patz, 1995). This approximation has been extensively validated and satisfies the fundamental assumption underlying Noise2Noise training. Accordingly, for the brain regions considered in this study, noisy MRI acquisitions can be treated as unbiased observations of the underlying anatomical structures.

Let x denote a low-resolution input image volume, and let y denote an acquired noisy high-resolution reference volume. The high-resolution reference can be expressed as $y=y*\;+n_{y}$, where y* represents the ideal noise-free high-resolution image volume and $n_{y}$ denotes zero-mean noise that is statistically independent of the input x. The goal is to learn a mapping function $f_{\theta }\left(x\right)$ parameterized by θ, by minimizing an L2 loss:

$$\begin{array}{c} \begin{array}{l} L\left(\theta \right)=E\left[{\left\|f_{\theta }\left(x\right)-y\;\right\|}^{2}\right]=E\left[{\left\|f_{\theta }\left(x\right)-y*\;-n_{y}\right\|}^{2}\right]\\ \;\;\;\;=E\left[{\left\|f_{\theta }\left(x\right)-y*\right\|}^{2}\right]+E\left[{\left\|n_{y}\right\|}^{2}\right]-2E\left[{\left(f_{\theta }\left(x\right)-y*\right)}^{T}n_{y}\right] \end{array} \end{array}$$ (1)

Because $n_{y}$ has zero mean and is statistically independent of both x and y* , the cross-term $E\left[{\left(f_{\theta }\left(x\right)-y*\right)}^{T}n_{y}\right]\;$ =0. Moreover, the noise variance term $E\left[{\left\|n_{y}\right\|}^{2}\right]$ is constant with respect to θ. Consequently, minimizing the loss using noisy targets y is statistically equivalent to minimizing $E\left[{\left\|f_{\theta }\left(x\right)-y*\right\|}^{2}\right]$, which corresponds to training with noise-free ground-truth image volumes. This equivalence provides the theoretical foundation for learning SR mappings using noisy high-resolution reference data. In addition, SRNR adopts a residual learning strategy, which further facilitates optimization and accelerates convergence.

### 2.2 Simulated data

To demonstrate the efficacy of SRNR and to evaluate its performance across different noise levels, we first performed experiments on simulated data. High-resolution T1-weighted MRI scans (0.45 × 0.45 × 0.9 mm³, interpolated from 0.9-mm isotropic acquisitions) were obtained from the publicly available Calgary Preschool MRI Dataset (Reynolds et al., 2020). Although the dataset underwent quality control before public release, visual inspection identified a small number of scans with noticeable artifacts. To ensure consistent image quality for simulation experiments, an additional automated quality assessment using MRIQC (Esteban et al., 2017) was performed. Scans with quality index $qi_{2}>0.0017$ were excluded, using thresholds determined through visual assessment by an experienced pediatric neuroradiologist. After quality control, 200 scans from 77 subjects were retained. To create independent cohorts with balanced age distributions, 60 scans from 21 subjects were randomly selected as the test set, whereas the remaining 140 scans from 56 subjects were used for training and validation. The retained scans were then resampled to 0.9-mm isotropic resolution using anti-aliasing Gaussian smoothing followed by linear interpolation.

To obtain anatomically realistic noise-free reference volumes, the T1-weighted image volume at 60 months from the Baby Connectome Project (BCP) atlas (0.8-mm isotropic resolution) (Chen et al., 2022) was registered to each subject’s T1-weighted image volume using the “SyNRA” registration framework implemented in ANTsPy (Tustison et al., 2021). SyNRA performs symmetric normalization through sequential rigid, affine, and deformable transformations optimized using mutual information, enabling the generation of subject-specific volumes with preserved anatomy and noise-free image contrast (Fig. 1A).

**Fig. 1.**
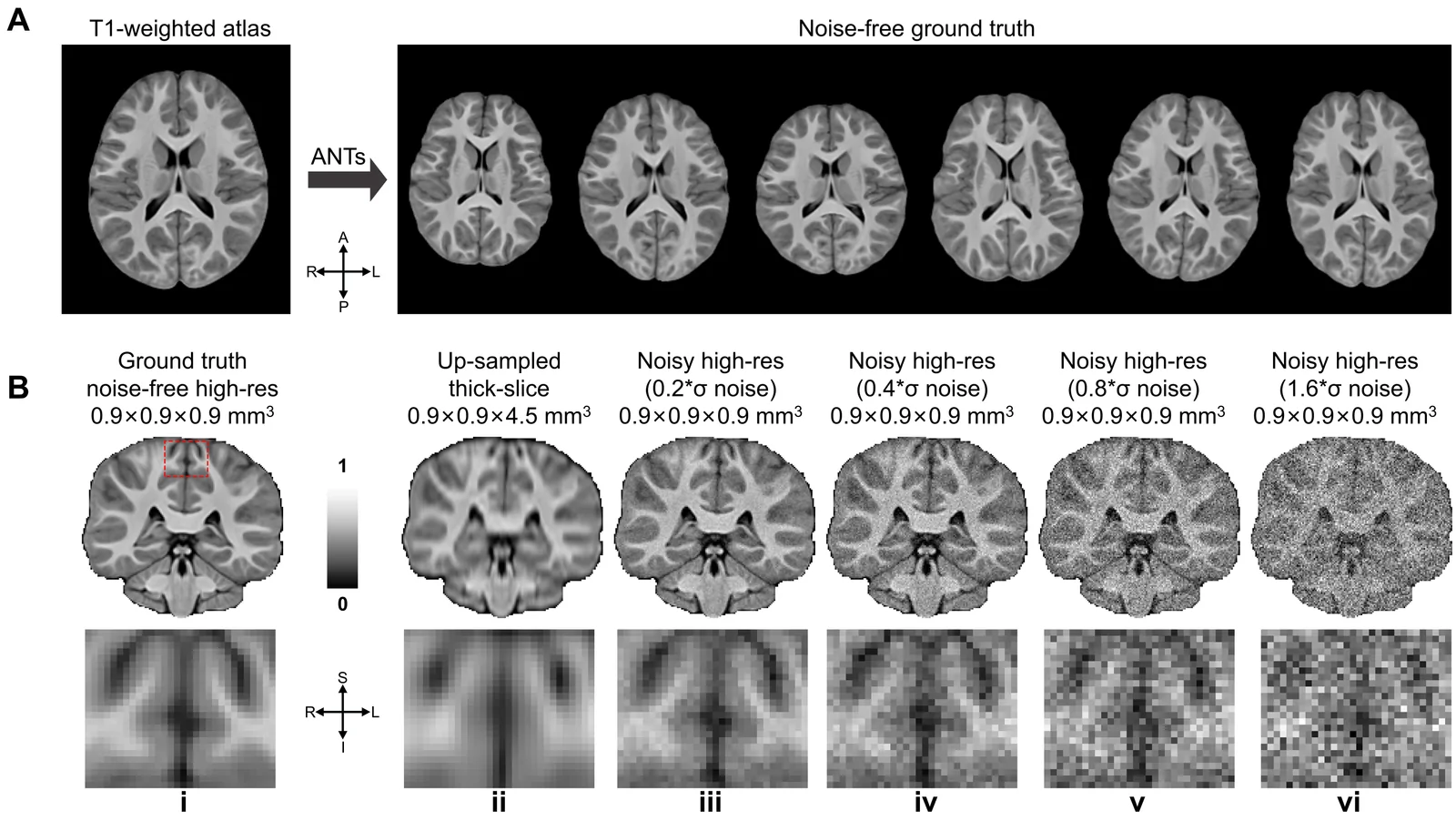
Generation of simulated data. (A) The T1-weighted image volume at 60 months from the Baby Connectome Project atlas was nonlinearly registered to each individual T1-weighted image volume from the publicly available Calgary Preschool MRI Dataset using the SyNRA registration framework implemented in ANTsPy, generating subject-specific, noise-free high-resolution ground-truth volumes. (B) Exemplar coronal slices from a representative subject in the independent test set are shown for the noise-free high-resolution ground truth (i), the up-sampled thick-slice input (ii), and simulated noisy high-resolution reference volumes (iii–vi). Gaussian noise was added with standard deviations set to 0.2σ (iii), 0.4σ (iv), 0.8σ (v), and 1.6σ (vi), where σ denotes the standard deviation of voxel intensities within the brain mask. The region of interest encompassing cortical gray and white matter, indicated by the red box, is shown in enlarged views.

Noisy high-resolution reference image volumes were generated by adding zero-mean Gaussian noise (μ = 0; σ = 0.2, 0.4, 0.8, 1.6 times the standard deviation of voxel intensities within brain masks) to the registered volumes (Fig. 1B). Corresponding low-resolution thick-slice image volumes with a spatial resolution of 0.9 × 0.9 × 4.5 mm³ were simulated by applying blurring followed by down-sampling along the superior–inferior axis, using a one-dimensional 48th-order Hamming-windowed low-pass filter. These low-resolution volumes were subsequently up-sampled to 1-mm isotropic resolution using cubic spline interpolation and served as the network inputs.

The detailed preprocessing procedures for the Human Connectome Project (HCP) dataset (Glasser et al., 2013, 2016) are described in Supplementary Note S1.

### 2.3 Empirical data

With institutional review board (IRB) approval, T1w MRI data were acquired from 12 healthy children on a 3T Siemens Skyra scanner equipped with a 32-channel receive coil at West China Second University Hospital. The participants ranged in age from 2 to 14 years (mean ± standard deviation: 8.1 ± 3.5 years). Both thick-slice, low-resolution T1w image volumes (3 × 1 × 1 mm³) and high-resolution T1w image volumes (1 × 1 × 1 mm³) were acquired using a prototype 3D Wave-CAIPI MPRAGE sequence (Ngamsombat et al., 2021; Polak et al., 2018, 2019) with identical core imaging parameters (Goncalves Filho et al., 2023; Longo et al., 2020): TR/TE/TI = 2,550/3.48/900 ms, flip angle = 8°, and bandwidth = 200 Hz/pixel. Thick-slice scans were acquired with an acceleration factor of R = 3 × 3 (30 seconds, one repetition), whereas high-resolution scans were acquired with R = 3 × 3 (66 seconds, one repetition) and R = 3 × 4 (51 seconds, one repetition) for each subject. All scans were performed in the sagittal orientation.

Brain masks were generated using the HD-BET algorithm (Isensee et al., 2019), which has demonstrated robust and reliable performance for brain extraction across heterogeneous MRI datasets, including pediatric populations. For image registration, the low-resolution T1w volumes were first up-sampled using trilinear interpolation and then aligned to the corresponding high-resolution T1w volumes (R = 3 × 3 or 3 × 4) using the NiftyReg software package (Modat et al., 2010, 2014). Due to the rapid acquisition of Wave-MPRAGE, large rigid displacements were unlikely. However, subtle non-rigid deformations and geometric distortions may arise from B_0_ inhomogeneity and gradient nonlinearity. To correct these residual spatial inconsistencies, a two-stage registration strategy was adopted. First, the NiftyReg function “reg_aladin” was used for global alignment based on a block-matching framework. The optimization was initialized with a rigid transformation and subsequently refined using a 12-degree-of-freedom affine model to correct residual global misalignment between the low- and high-resolution volumes. Subsequently, the “reg_f3d” function was applied for non-rigid registration using a cubic B-spline free-form deformation model, enabling correction of local anatomical deformations and nonlinear discrepancies to further improve spatial alignment and anatomical correspondence.

To establish a high-SNR benchmark for model evaluation, the high-resolution T1w reference image volumes were denoised using BM4D (Maggioni et al., 2013). Because accelerated acquisitions introduce substantial noise, directly using raw image volumes as references would bias SR evaluation, particularly for noise-sensitive downstream analyses such as cortical morphometry and anatomical localization (Lüsebrink et al., 2013). The noise standard deviation was estimated from the whole brain using a wavelet-based robust estimator (Donoho & Johnstone, 1994). The estimated σ was multiplied by a factor determined through grid search on the validation data, with candidate multipliers ranging from 1.0 to 3.0. A multiplier of 2.5 achieved the optimal trade-off between noise suppression and preservation of cortical anatomical details. The resulting noise level estimate was then provided to BM4D with the normal profile to generate denoised references approximating high-SNR clinical-quality images. The denoised references were used exclusively for model evaluation and were not used as targets during model training.

### 2.4 Network architecture and training

SRNR is a general training paradigm that enables a wide range of neural networks to learn from noisy high-resolution references without requiring clean targets. In this study, a modified U-Net (MU-Net; Fig. 2) (Li et al., 2022) was employed as $f_{\theta }$. To preserve fine spatial details and minimize blurring, all max-pooling and up-sampling layers in the conventional U-Net were removed, resulting in a fully convolutional architecture without resolution-altering operations. Residual learning was incorporated to improve training robustness and accelerate convergence, a strategy widely adopted in SR frameworks for its effectiveness in simplifying the learning task and improving image fidelity (Kim et al., 2016; Tian et al., 2020; Zhang et al., 2017). The MU-Net took the low-resolution T1w volume up-sampled to the target resolution as input, and the predicted residual was added to the input to produce the final super-resolved image volume.

**Fig. 2.**
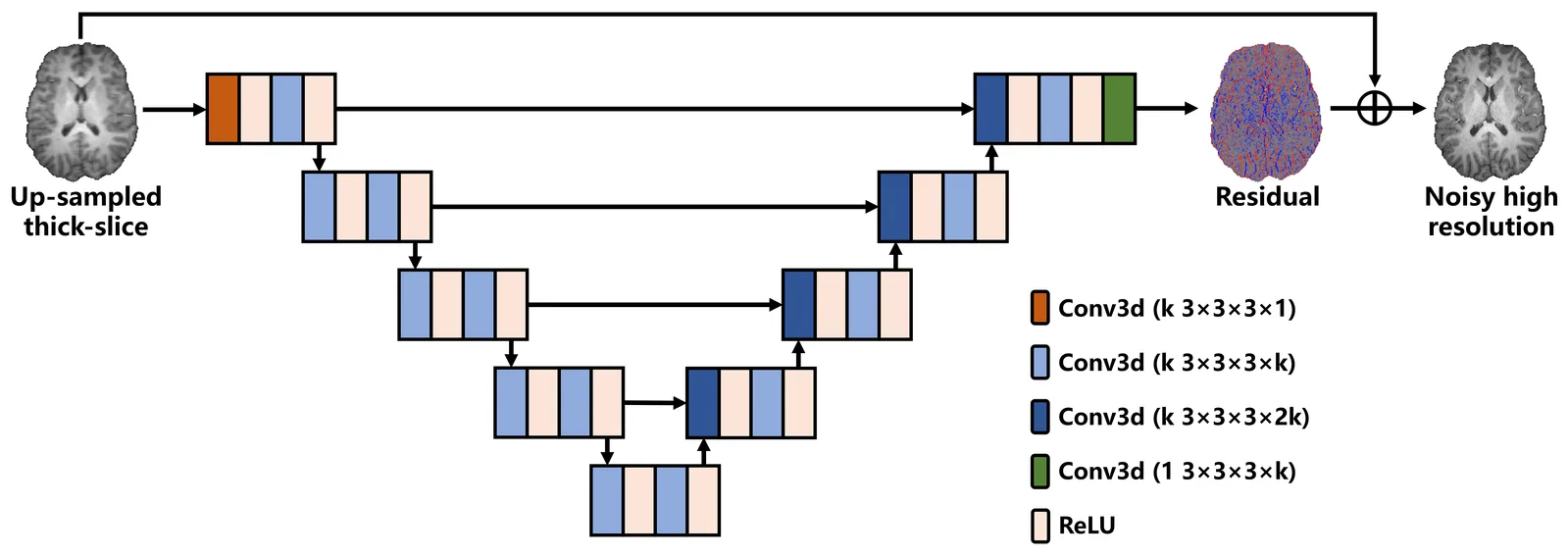
Neural network architecture. A modified 3D U-Net (MU-Net) composed of 3D convolutional layers, ReLU activations, and skip connections, with max-pooling and up-sampling layers removed to better preserve spatial details, is adopted. MU-Net predicts the residual between the up-sampled low-resolution image volume and the target high-resolution image volume. The final output is obtained by adding the predicted residual back to the input image volume.

For the simulation experiments, 140 co-registered noise-free image volumes were used for training and validation, while an independent set of 60 image volumes was held out for testing. Overlapping 3D patches of 96 × 96 × 96 voxels were extracted using a deterministic sliding window covering the bounding box of the brain mask. Both input and target volumes were Z-score normalized within the brain mask using the mean and standard deviation calculated from the input volume. No spatial augmentation was applied. The network was configured with 64 feature channels and optimized using Adam with a brain-mask-weighted mean squared error (MSE) loss (Kingma & Ba, 2017). Within the training and validation cohort, patches were randomly divided into training and validation sets at a ratio of 80%/20%. Training was performed for 50 epochs with a batch size of 1. The learning rate was initialized at 1 × 10^−^³ and reduced to 3 × 10^−4^ and 1 × 10^−4^ at epochs 15 and 35, respectively.

For the empirical experiments, data from eight pediatric subjects were used for training and validation, while four subjects were held out for testing. Following the same preprocessing and patch extraction strategy as in the simulation experiments, left–right flipping was additionally applied for data augmentation. The network was configured with 128 feature channels and optimized using a fixed learning rate of 1 × 10^−4^, while all other training settings remained unchanged.

During inference, overlapping patch predictions were aggregated by voxel-wise averaging to reconstruct the whole-brain residual volume, which was then added to the up-sampled input to generate the final super-resolved output. All preprocessing and model training were implemented in TensorFlow v2.6.0 and performed on a workstation equipped with two Intel Xeon CPUs and one NVIDIA A800 GPU.

### 2.5 Transfer learning

To evaluate the feasibility of fine tuning with a very limited amount of noisy clinical data for supervision, a transfer learning strategy was employed. The simulated data were first reprocessed using the same down-sampling factor (×3) applied to the pediatric clinical acquisitions to reduce domain mismatch. To better match the noise characteristics of real clinical data, a network with 64 channels was first pre-trained on simulated data with a low noise level (0.4 × σ) using a fixed learning rate of 0.001. Subsequently, fine tuning was performed using only 3 pediatric clinical cases for a total of 20 epochs. The first 10 epochs used a learning rate of 0.0001, followed by another 10 epochs with a learning rate of 0.00005.

### 2.6 Ablation study

To strictly isolate the effect of pooling and up-sampling on network performance, we constructed a parameter-matched conventional 3D U-Net baseline. The conventional U-Net and MU-Net had identical convolutional depth and parameter capacity, comprising 9 convolutional stages organized into 5 encoding and 4 decoding stages, with 64 feature channels in each stage. The only architectural difference between the two networks was their spatial sampling strategy. The conventional U-Net incorporated four 2 × 2 × 2 3D max-pooling layers for spatial compression and corresponding up-sampling operations for feature resolution restoration. In contrast, MU-Net removed all pooling and up-sampling operations and maintained a single-scale, full-resolution feature stream throughout the network. Both networks were trained using the same SRNR paradigm, training data, data splits, and optimization settings.

### 2.7 Comparison methods

SRNR was compared with SMORE (Zhao et al., 2021), a representative scan-specific self-supervised MRI super-resolution method that does not require external training data. SMORE constructs self-supervised training pairs from the high-resolution in-plane information available within each anisotropic input and learns to recover high-resolution information along the through-plane direction, generating isotropic volumes with the in-plane spatial resolution. The publicly available implementation and default configuration provided by the SMORE developers were adopted. Following the recommended workflow, subject-specific adaptation was performed for each test volume using the single thick-slice input, and the adapted model was subsequently applied to the corresponding input volume to generate the super-resolved reconstruction.

### 2.8 Downstream tasks

To further evaluate the practical utility of SRNR, downstream analyses, including cortical surface reconstruction and volumetric segmentation, were performed using the standard FreeSurfer (Fischl, 2012) pipeline, and the task performance was compared before and after SR.

Cortical surface reconstruction was conducted using FreeSurfer to generate the gray–white matter interface and the gray–CSF interface, both of which are critical for whole-brain morphometric analyses. Cortical thickness and surface positioning were quantified using the FreeSurfer longitudinal processing pipeline (Reuter & Fischl, 2011; Reuter et al., 2010, 2012), which estimates the spatial positions of both interfaces across the cortex. Volumetric segmentation accuracy was quantified using FreeSurfer’s “aparc+aseg” results, which label a comprehensive set of cortical and subcortical brain structures.

### 2.9 Evaluation

To comprehensively assess the difference between super-resolved image volumes and the high-resolution references, whole-brain-averaged mean absolute error (MAE), peak signal-to-noise ratio (PSNR), and structural similarity index (SSIM) were computed. These metrics quantify image similarity, reconstruction fidelity, and the ability to recover fine anatomical details. All metrics were calculated within the brain mask for up-sampled image volumes, as well as for super-resolved outputs generated by networks trained with reference data at varying noise levels, using the high-SNR, high-resolution image volumes as the reference. In addition, SRNR outputs were compared with those obtained from networks trained using high-SNR reference data, enabling a direct assessment of the impact of noisy supervision on SR performance.

For volumetric segmentation, Dice similarity coefficients were computed between the segmentations derived from super-resolved image volumes and the standard high-resolution reference image volumes. Dice similarity coefficients were evaluated for 12 key brain structures, including cerebral white matter, cerebral cortex, hippocampus, and brainstem. Group-level means and standard deviations were reported to quantify segmentation consistency and to assess the anatomical fidelity of the super-resolved image volumes.

For cortical surface reconstruction, vertex-wise discrepancies in surface positioning and cortical thickness were quantified by comparing results derived from the up-sampled and super-resolved image volumes against the high-resolution reference. To evaluate cortical thickness accuracy, group-level means and standard deviations of the whole-cortex-averaged absolute differences in surface position and cortical thickness were computed.

In addition, a widely used no-reference metric, the tissue contrast t-score (TCT) (Pizarro et al., 2016; Sun et al., 2024), was employed to quantify gray matter (GM)–white matter (WM) contrast and to complement the reference-based evaluation metrics. TCT was calculated for each image volume based on the segmented GM and WM regions to assess the effect of SR reconstruction on tissue contrast. TCT is defined as

$$TCT=\frac{\left|\mu_{GM}-\mu_{WM}\right|}{\sqrt{\sigma_{GM}^{\;\;\;\;\;\;\;\;\;\;2}+\sigma_{WM}^{\;\;\;\;\;\;\;\;\;\;2}}},$$ (2)

where $\mu_{GM}$ and $\mu_{WM}$ denote the mean intensities of GM and WM, respectively, and $\sigma_{GM}$ and $\sigma_{WM}$ denote their corresponding standard deviations. Higher TCT values indicate stronger GM–WM contrast and more uniform intensity distributions within each tissue.

## 3 Results

### 3.1 Simulation results

Simulation experiments demonstrated that SRNR produced super-resolved image volumes that were visually highly consistent whether the networks were trained using high-SNR or noisy high-resolution references (Fig. 3a). Compared with up-sampled low-resolution inputs, SRNR outputs exhibited markedly enhanced textural and structural detail and sharper tissue boundaries. Enlarged views further showed well-preserved gray–white matter contrast and spatial organization consistent with the ground-truth image volumes (Fig. 3b). Importantly, increasing noise levels in the training references had no appreciable impact on the quality of the super-resolved outputs (Fig. 3a, iii–vii). Even when the noisiest high-resolution references were used for training, the resulting super-resolved image volumes remained visually indistinguishable from those obtained using noise-free ground truths (Fig. 3a, iii vs. Fig. 3a, vii). This observation is consistent with the theoretical expectation that SRNR converges to clean, high-resolution outputs even when trained with noisy reference data. Accordingly, residual maps between the super-resolved and ground-truth image volumes contained little structured anatomical information and exhibited substantially smaller errors than those observed for the up-sampled inputs (Fig. 3c, d).

**Fig. 3.**
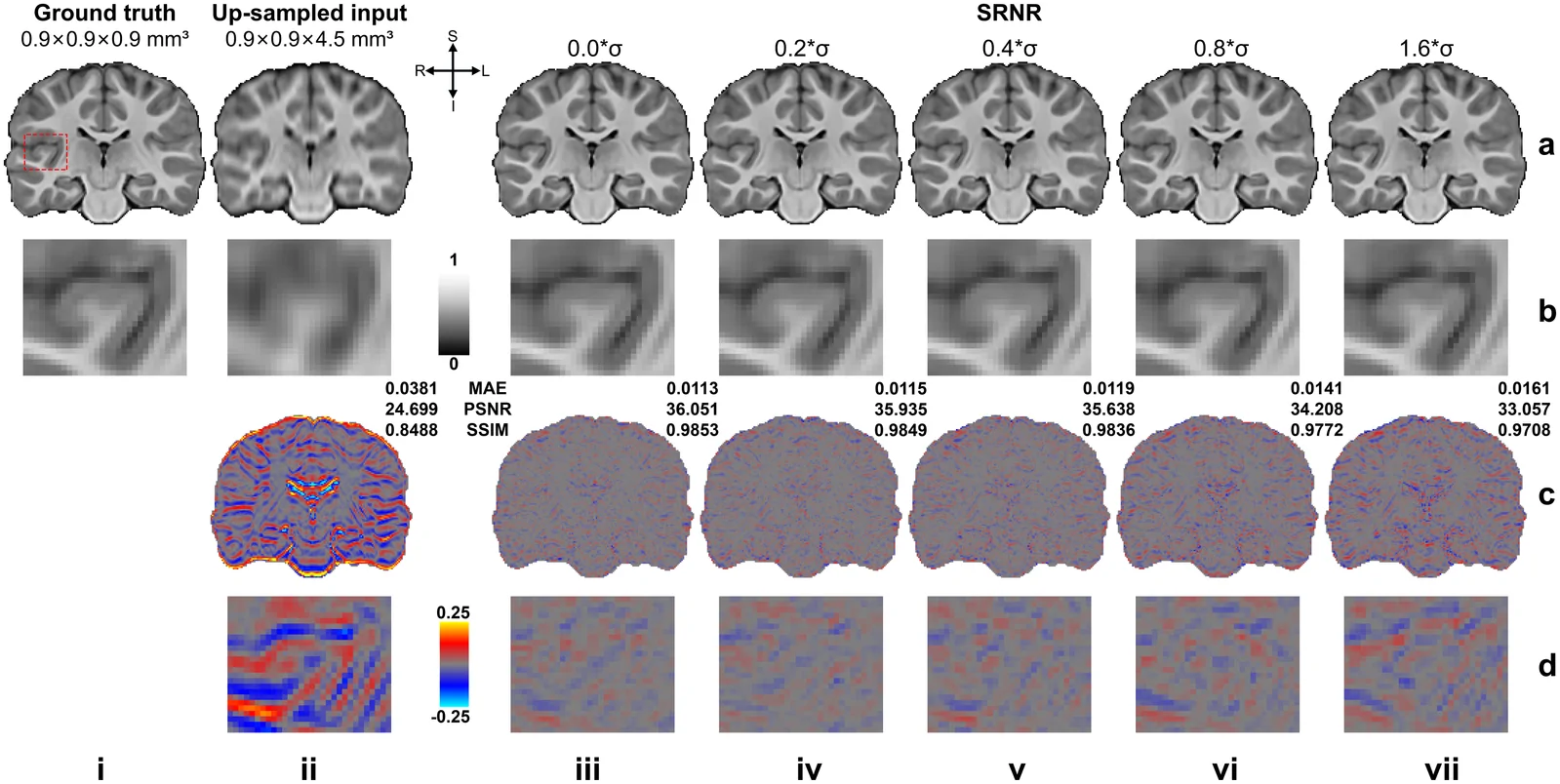
Simulation experiments across training-target noise levels. Exemplar coronal slices from a representative participant in the independent test set from the publicly available Calgary Preschool MRI Dataset are shown for the ground-truth high-resolution image volume (a, i), the up-sampled low-resolution input (a, ii), the super-resolved prediction from a neural network trained using noise-free high-resolution references (a, iii), and SRNR predictions from networks trained using simulated noisy high-resolution references (a, iv–vii; with noise standard deviation equal to 0.2 (iv), 0.4 (v), 0.8 (vi), and 1.6 (vii) times the standard deviation of voxel intensities within the brain mask). The region of interest encompassing cortical gray and white matter, indicated by the red box, is shown in enlarged views (b). Whole-brain difference maps (c) and corresponding enlarged views (d) depict the voxel-wise differences between the up-sampled or super-resolved image volumes and the ground truth. The whole-brain mean absolute error (MAE), peak signal-to-noise ratio (PSNR), and structural similarity index (SSIM) for the exemplar participant are reported to quantify image similarity.

Quantitative analysis further confirmed that SRNR significantly improved super-resolved image quality relative to up-sampled inputs and achieved strong agreement with the ground truths across all noise conditions (Fig. 4A, C). Specifically, SRNR reduced the mean MAE from 0.038 to 0.012–0.017 and increased the mean PSNR from 24.791 to 32.496–35.250 dB, as well as the mean SSIM from 0.848 to 0.966–0.982 (two-sided paired *t*-test, *P* < 0.001). As noise levels increased, image similarity between the noisy training references and the ground truths deteriorated markedly, following an approximately linear decline (Fig. 4B). In contrast, the similarity of SRNR super-resolved predictions exhibited only a modest reduction. Even under the highest noise condition (1.6 × σ), performance degradation remained limited, with changes of only 38% in MAE, 2.75 dB in PSNR, and 1.6% in SSIM relative to the high-SNR setting. Moreover, super-resolved predictions obtained using noisy and noise-free training references were highly consistent (MAE: 0.010–0.014; PSNR: 34.331–37.293 dB; SSIM: 0.978–0.989; Fig. 4D), indicating that training with noisy reference volumes introduced negligible performance loss.

**Fig. 4.**
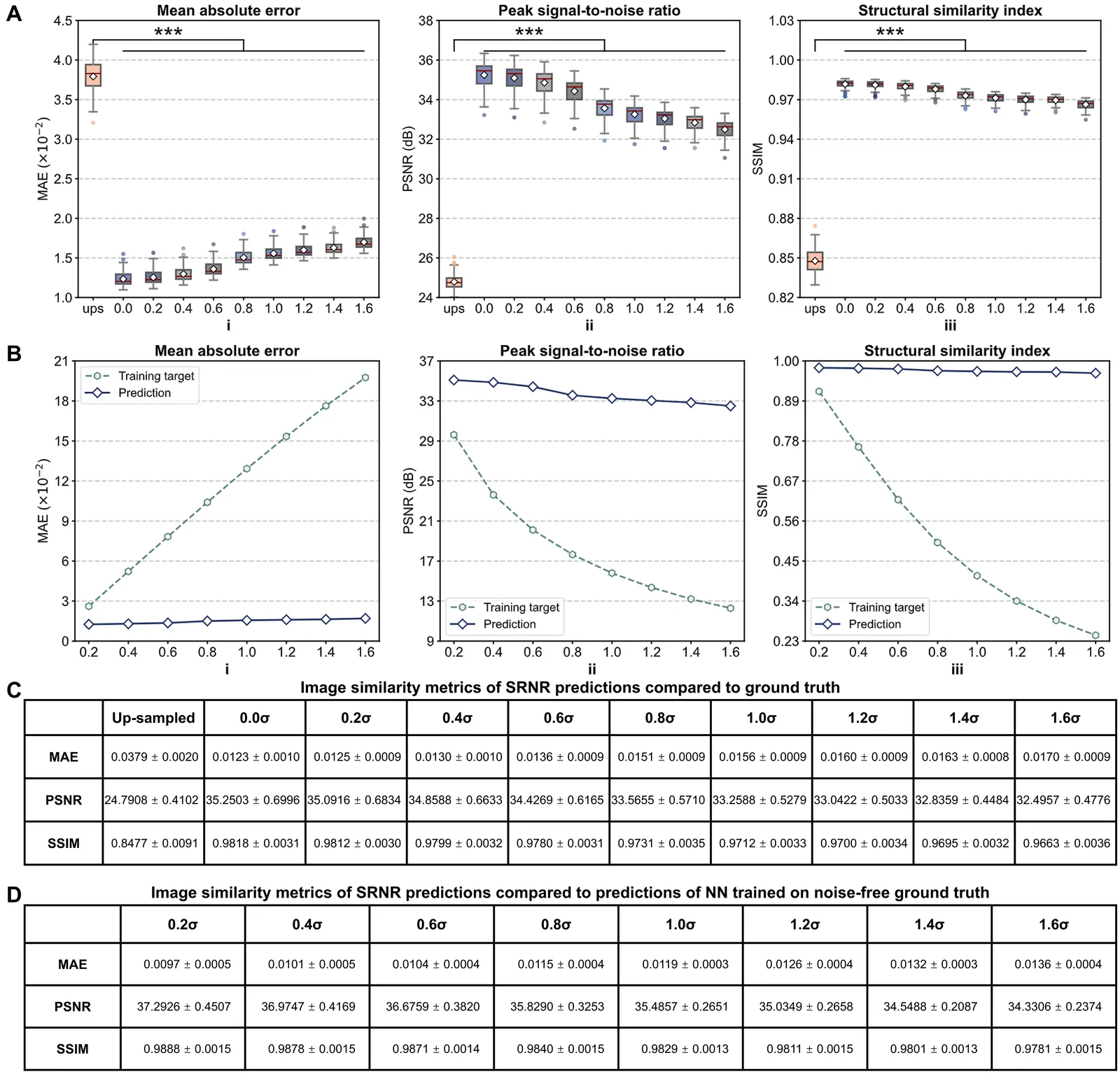
Image similarity metrics of simulation experiments. (A) Box plots illustrate the whole-brain-averaged MAE (i), PSNR (ii), and SSIM (iii) across 60 evaluation scans. Metrics were computed for the up-sampled low-resolution image volumes (ups) and for super-resolved outputs from neural networks trained on noise-free references (i.e., 0.0σ) and on simulated noisy high-resolution references (with noise standard deviations ranging from 0.2 to 1.6 times the standard deviation of voxel intensities within the brain mask), all reported relative to the noise-free ground-truth high-resolution volumes. The midline indicates the median, and box edges indicate the first and third quartiles. Whiskers extend to the most extreme observations within 1.5 times the interquartile range, and observations beyond the whiskers are shown individually. Statistical significance for comparisons between up-sampled and super-resolved outputs is indicated by asterisks (^***^P < 0.001, two-sided paired *t*-test, *n* = 60). (B) Line plots show the group mean MAE (i), PSNR (ii), and SSIM (iii) of the simulated noisy training references (green dashed lines) and the corresponding super-resolved outputs (blue solid lines) across varying noise levels, both calculated relative to the ground truths. (C) Group means (± standard deviations) of the quantitative values shown in A are listed. (D) Group means (± standard deviations) of whole-brain-averaged MAE, PSNR, and SSIM for super-resolved outputs from neural networks trained with noisy high-resolution references, reported relative to those from neural networks trained with noise-free references, across 60 evaluation scans, are listed.

Volumetric segmentation of cerebral WM and cerebral cortex derived from SRNR-reconstructed simulated data exhibited high accuracy. Across training references with varying noise levels, SRNR consistently produced segmentation results that were visually comparable with the ground truths and demonstrated finer anatomical detail than those obtained from up-sampled image volumes (Fig. 5A). In localized regions, SRNR reconstructions corrected structural errors observed in the up-sampled segmentations. Quantitatively, perceptual tissue contrast in the super-resolved predictions, as measured by the TCT, was substantially improved (6.345–6.479) relative to the up-sampled image volumes (3.484) and approached that of the ground truths (6.470). Dice similarity coefficients were also significantly higher for super-resolved outputs than for up-sampled inputs across both structures (cerebral WM Dice: 0.951–0.960 vs. 0.807; cerebral cortex Dice: 0.935–0.947 vs. 0.770; average Dice: 0.943–0.954 vs. 0.789; two-sided *t*-test, *P* < 0.001; Fig. 5B). Increasing noise levels in the training references led to only a marginal reduction in segmentation performance. Dice similarity coefficients for all 12 individual brain structures and for all structures combined are reported in Supplementary Table S1.

**Fig. 5.**
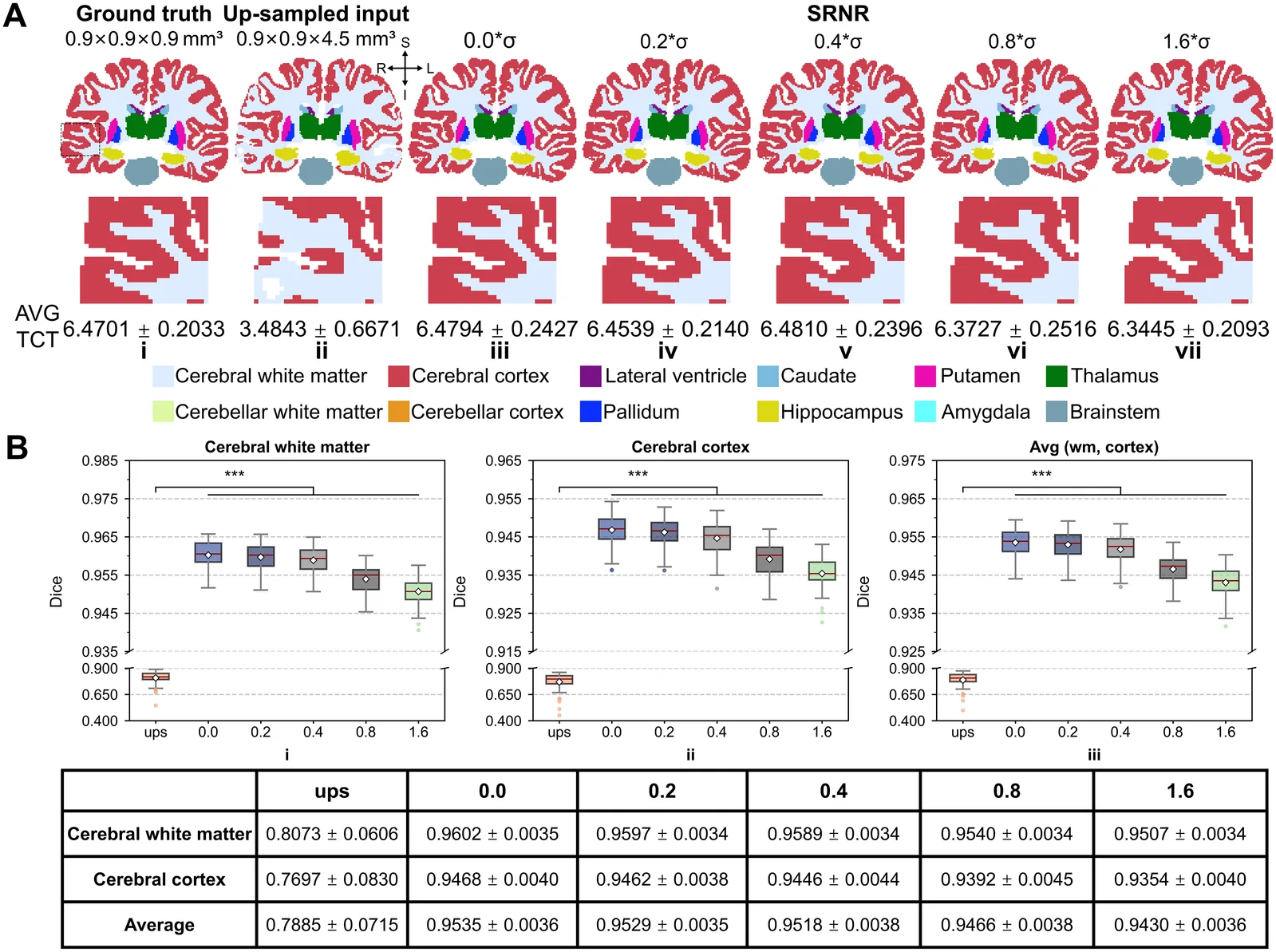
Volumetric segmentation of simulation experiments. (A) Exemplar coronal slices from a representative subject in the independent test set illustrate segmented brain structures derived from the high-resolution, noise-free ground-truth image volume (i), the up-sampled thick-slice image volume (ii), and super-resolved outputs from neural networks trained using high-resolution reference data with varying noise levels (iii–vii). The region of interest encompassing cortical gray and white matter, indicated by the black box, is shown in enlarged views. Average tissue contrast *t*-score (TCT) values are also provided. (B) Box plots show scan-level Dice similarity coefficients across 59 evaluation scans (1 subject was excluded due to cortical reconstruction failure in the up-sampled volume) for segmentations derived from up-sampled thick-slice image volumes (ups) and SRNR super-resolved outputs, evaluated for cerebral white matter (i), cerebral cortex (ii), and their average (iii). White diamonds indicate the means, horizontal lines within the boxes indicate the medians, and box boundaries indicate the first and third quartiles. Whiskers extend to the most extreme observations within 1.5 times the interquartile range, and observations beyond the whiskers are shown individually. All SRNR noise-level conditions differed significantly from ups in each panel (^***^P < 0.001, two-sided paired *t*-test, *n* = 59). Dice similarity coefficients for all 12 individual brain structures and for all structures combined are reported in Supplementary Table S1.

SRNR further improved cortical surface reconstruction accuracy, resulting in more accurate brain morphometric measurements (Fig. 6). Although cortical surfaces reconstructed from up-sampled low-resolution image volumes preserved the overall geometry of the ground truths, they exhibited distortions in fine structural details. In contrast, super-resolved outputs generated by SRNR showed improved surface continuity and clearer delineation of gyral and sulcal boundaries, yielding reconstructions that were smoother and more consistent with the high-resolution references. These improvements were further corroborated by quantitative analyses. Compared with up-sampled image volumes, super-resolved outputs achieved lower errors in cortical thickness estimation (0.200–0.255 vs. 0.715), gray–white matter boundary positioning (0.321–0.366 vs. 0.791), and gray–CSF boundary positioning (0.240–0.279 vs. 0.631). Importantly, these gains were preserved across increasing noise levels in the training references, indicating robust morphometric performance of SRNR under noisy supervision.

**Fig. 6.**
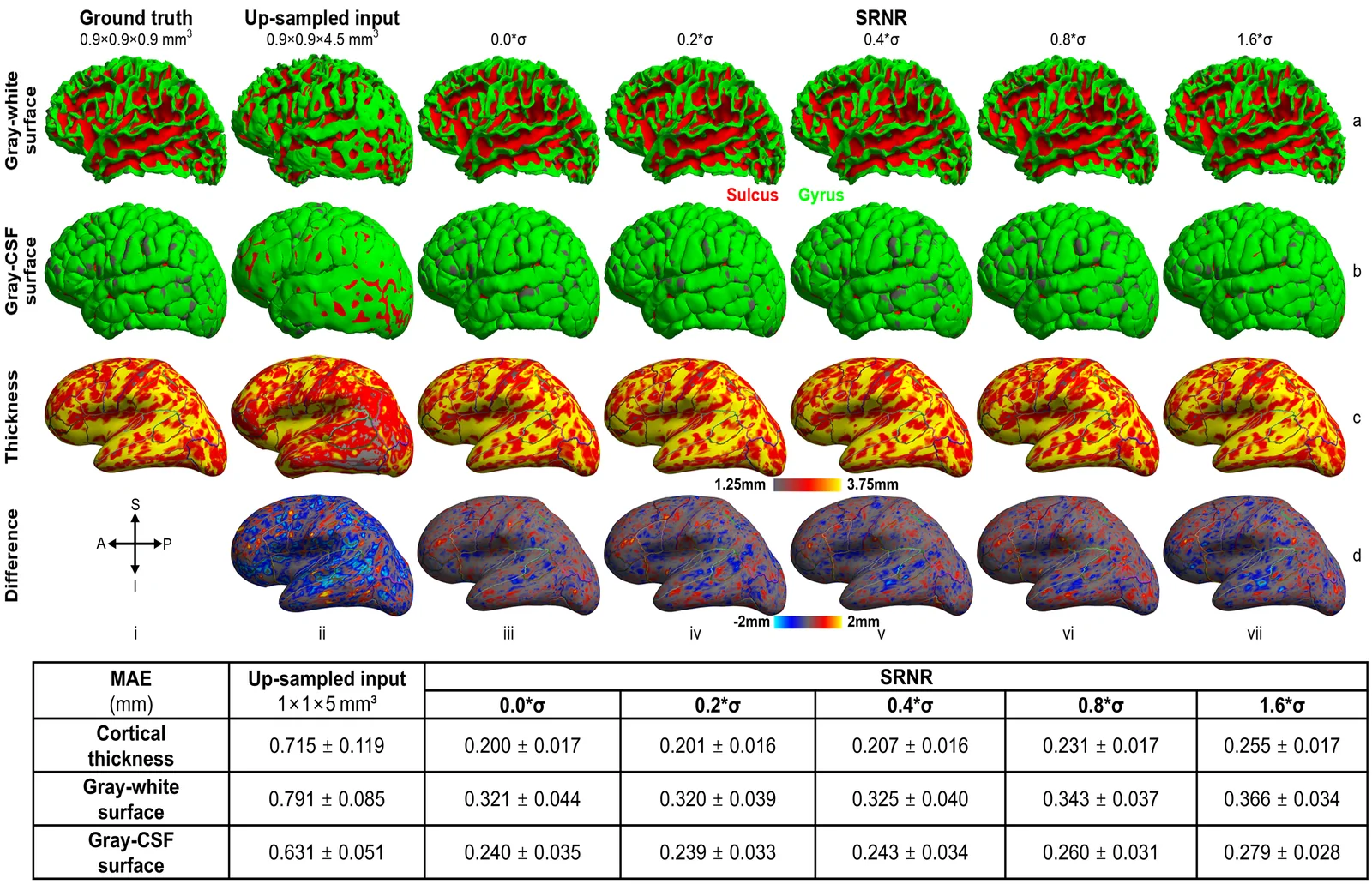
Cortical surface reconstruction and cortical thickness accuracy of simulation experiments. Cortical surface reconstruction results are shown for a representative subject from the independent test set, including gray–white matter interface surfaces (a) and gray–CSF interface surfaces (b) reconstructed from the high-resolution, noise-free ground-truth image volume (i), the up-sampled thick-slice image volume (ii), and super-resolved outputs (iii–vii) from neural networks trained using high-resolution reference volumes with varying noise levels. Left-hemispheric vertex-wise cortical thickness (c) and its difference relative to the ground truth (d) are displayed on the inflated surface. Different cortical regions defined by the FreeSurfer cortical parcellation (i.e., aparc.annot) are depicted as colored outlines. Group means (± standard deviations) of the whole-brain-averaged absolute differences in cortical thickness and the displacements of the gray–white and gray–CSF surfaces estimated from the up-sampled and super-resolved outputs relative to the ground truth are reported.

In addition to the pediatric dataset, we further validated SRNR using simulated experiments based on the adult Human Connectome Project (HCP) dataset (Glasser et al., 2013, 2016). The results demonstrated that SRNR achieved comparable performance and consistent findings on adult brain MRI data. Specifically, across varying noise levels in the training references, SRNR consistently produced high-quality super-resolved outputs with mean PSNR values ranging from 27.093 to 27.824 dB and SSIM values from 0.912 to 0.925 (Supplementary Figs. S1–S3). These quantitative improvements were accompanied by enhanced downstream task performance: volumetric segmentation showed substantially higher Dice coefficients for cerebral WM (0.911–0.922 vs. 0.896 for up-sampled inputs) and cerebral cortex (0.848–0.867 vs. 0.771), while cortical surface reconstruction exhibited reduced errors in both surface positioning and cortical thickness estimation (Supplementary Figs. S4–S5 and Supplementary Table S3). These findings indicate that SRNR maintains consistent performance across different age groups and can be applied to diverse neuroimaging applications beyond pediatric populations.

### 3.2 Ablation and method comparison

To evaluate the effect of removing the max-pooling and up-sampling operations, we conducted an ablation study by comparing MU-Net with a conventional U-Net while keeping the SRNR training paradigm, training data, and optimization settings unchanged (Fig. 7). Qualitatively, both architectures generated super-resolved image volumes that closely matched the ground-truth volumes. However, MU-Net better preserved fine anatomical structures, exhibiting sharper tissue boundaries and fewer residual errors. Quantitatively, removing the spatial resolution-changing operations reduced the mean MAE from 0.0134 to 0.0130 while increasing the mean PSNR from 34.5735 to 34.8588 dB and the mean SSIM from 0.9789 to 0.9799. These results indicate that preserving the spatial resolution of intermediate feature maps provides a modest but consistent improvement in reconstruction fidelity. Nevertheless, the relatively small performance difference between the two architectures suggests that the primary performance gains originate from the proposed SRNR training paradigm rather than the architectural modification itself, indicating that SRNR can be applied across different super-resolution network architectures.

**Fig. 7.**
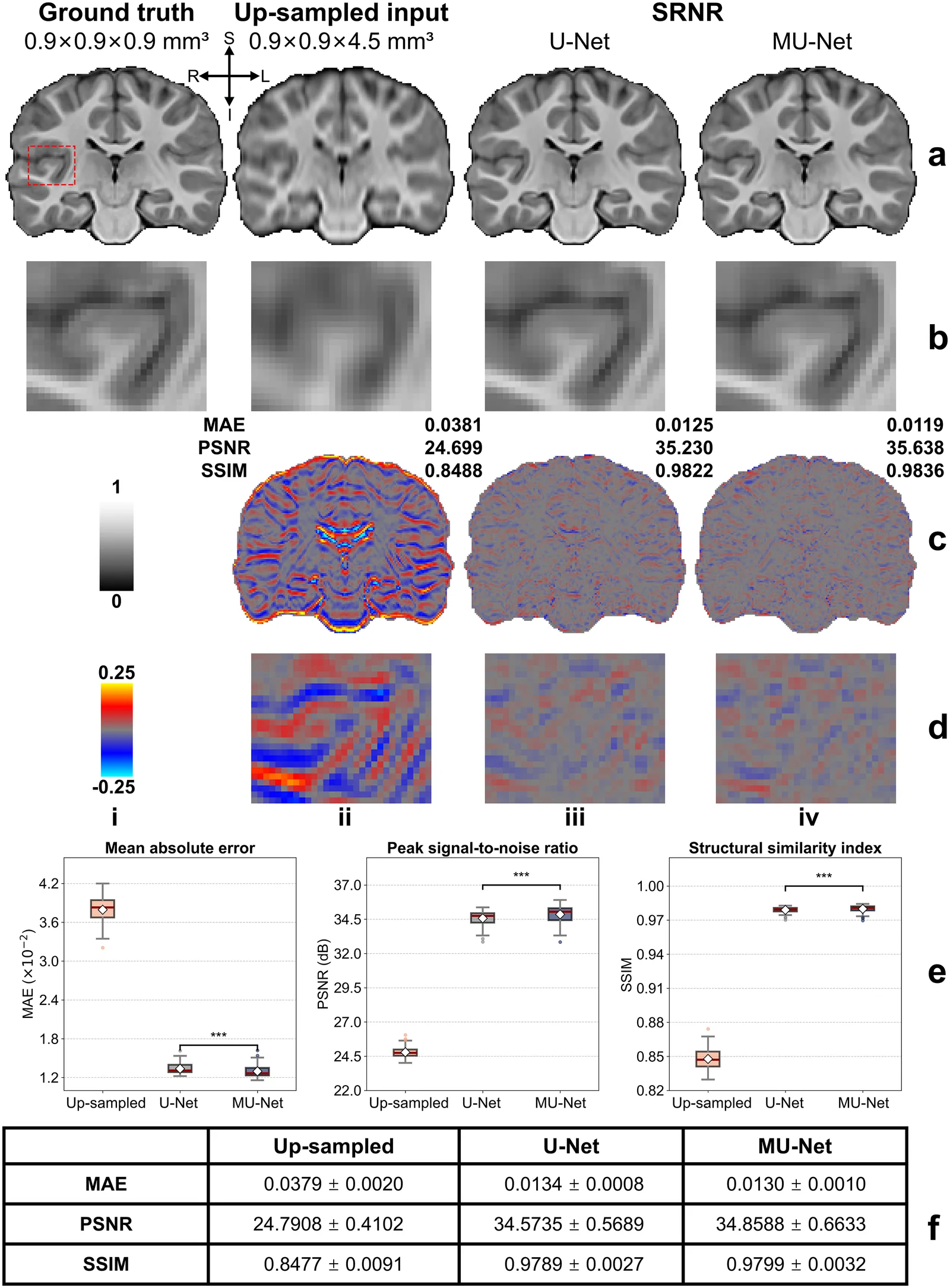
Comparison of image reconstruction quality between MU-Net and U-Net in simulation experiments. Exemplar coronal slices from a representative subject in the independent test set show the ground-truth high-resolution image volume (a, i), the up-sampled thick-slice input (a, ii), and super-resolved predictions obtained using a conventional U-Net (a, iii) and MU-Net (a, iv), both trained using the SRNR paradigm under identical conditions with simulated noisy high-resolution references (0.4σ). The region of interest encompassing cortical gray and white matter, indicated by the red box, is shown in enlarged views (b). Whole-brain difference maps (c) and corresponding enlarged views (d) depict voxel-wise differences between the up-sampled or super-resolved image volumes and the ground truth. MAE, PSNR, and SSIM for the representative subject are listed. Box plots show the corresponding whole-brain-averaged metrics across 60 held-out test volumes for the up-sampled inputs and the SRNR predictions obtained using U-Net and MU-Net (e). The horizontal line within each box indicates the median, diamond markers denote the mean, and box edges indicate the first and third quartiles. Whiskers extend to the most extreme observations within 1.5 times the interquartile range, and observations beyond the whiskers are shown individually. MU-Net significantly outperformed U-Net in each metric (^***^P < 0.001, two-sided paired *t*-test, *n* = 60). Group means ± standard deviations of the corresponding metrics are listed (f).

Comparison with the representative self-supervised MRI super-resolution method SMORE further demonstrated the effectiveness of SRNR (Fig. 8). Qualitatively, SRNR generated image volumes that most closely matched the ground-truth volumes, exhibiting sharper tissue boundaries, clearer cortical details, and substantially fewer structured residuals in the difference maps. Although SMORE improved image quality relative to the up-sampled inputs, it retained visible blurring and larger residual errors. Quantitatively, compared with SMORE, SRNR reduced the mean MAE from 0.0314 to 0.0130 while improving the mean PSNR from 26.7093 to 34.8588 dB and the mean SSIM from 0.8941 to 0.9799, demonstrating substantially more accurate recovery of high-resolution anatomical details.

**Fig. 8.**
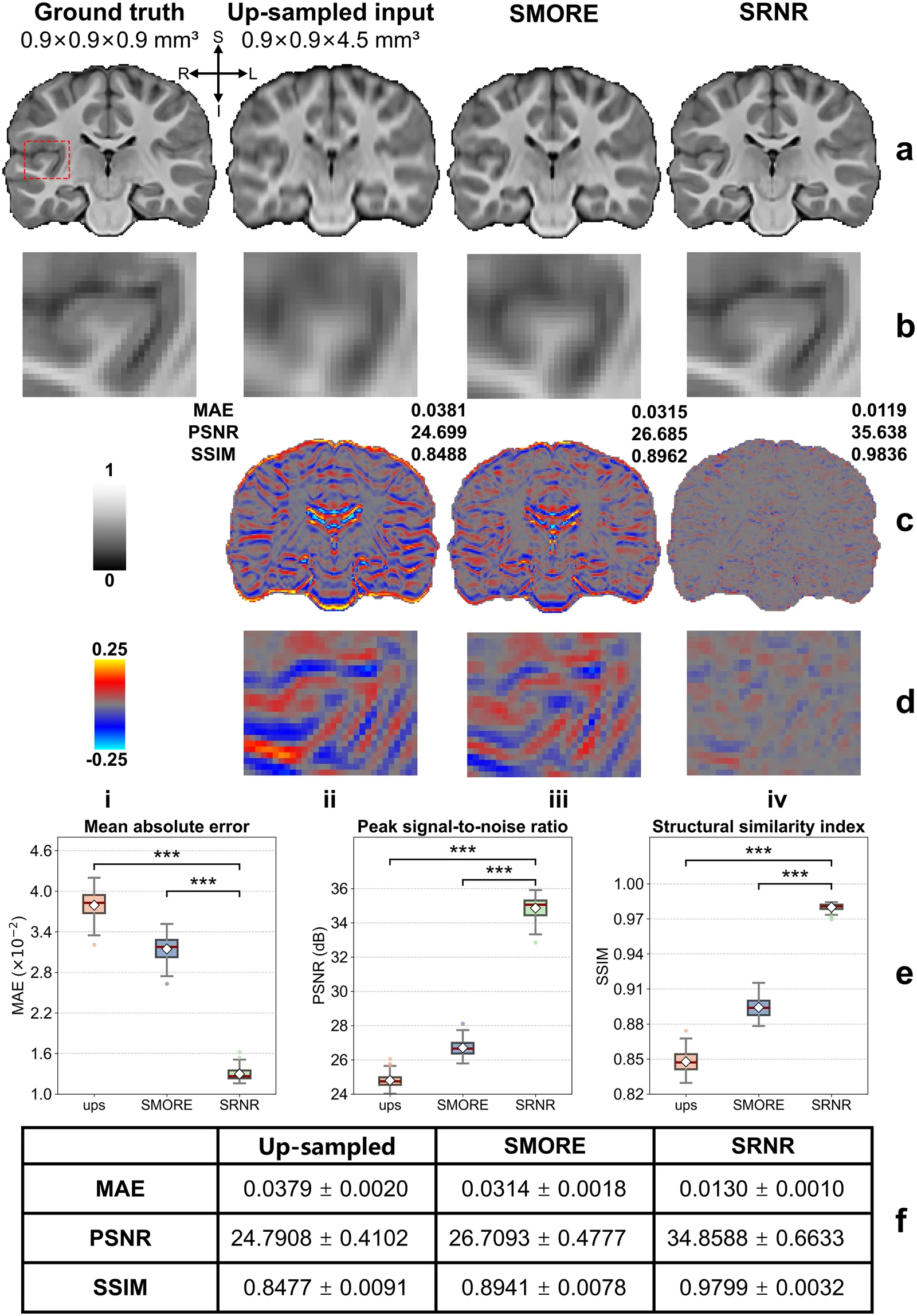
Comparison of image reconstruction quality between SRNR and SMORE in simulation experiments (0.4σ). Exemplar coronal slices from a representative subject in the independent test set show the ground-truth high-resolution image volume (a, i), the up-sampled (ups) thick-slice input (a, ii), the super-resolution prediction generated by SMORE (a, iii) and SRNR (a, iv). The region of interest encompassing cortical gray and white matter, indicated by the red box, is shown in enlarged views (b). Whole-brain difference maps and corresponding enlarged views depict voxel-wise differences between each reconstructed image volume and the ground truth. Whole-brain-averaged MAE, PSNR, and SSIM for the representative subject are listed (c, d). Box plots show the whole-brain-averaged MAE, PSNR, and SSIM across the independent test set for the up-sampled inputs and predictions from the two methods. The midline indicates the median, diamond markers denote the mean, box edges indicate the first and third quartiles, and whiskers represent the minimum and maximum values (e). SRNR significantly outperformed up-sampled and SMORE in each metric (^***^P < 0.001, two-sided paired *t*-test, *n* = 60). Group means ± standard deviations of the corresponding metrics are listed (f).

Consistent with the image-level comparisons, SRNR also improved downstream segmentation performance (Fig. 9). Qualitatively, segmentations derived from SRNR most closely matched those derived from the ground-truth images, particularly in preserving cortical boundaries and fine anatomical details. Quantitatively, SRNR achieved a mean TCT of 6.4769, closely matching that of the ground-truth measurement (6.4745) and substantially outperforming those of the up-sampled inputs (3.4731) and SMORE (4.8933). Similarly, compared with SMORE, SRNR improved the Dice similarity coefficient for cerebral WM from 0.8891 to 0.9589 and for cerebral cortex from 0.8605 to 0.9447, increasing the average Dice from 0.8748 to 0.9518.

**Fig. 9.**
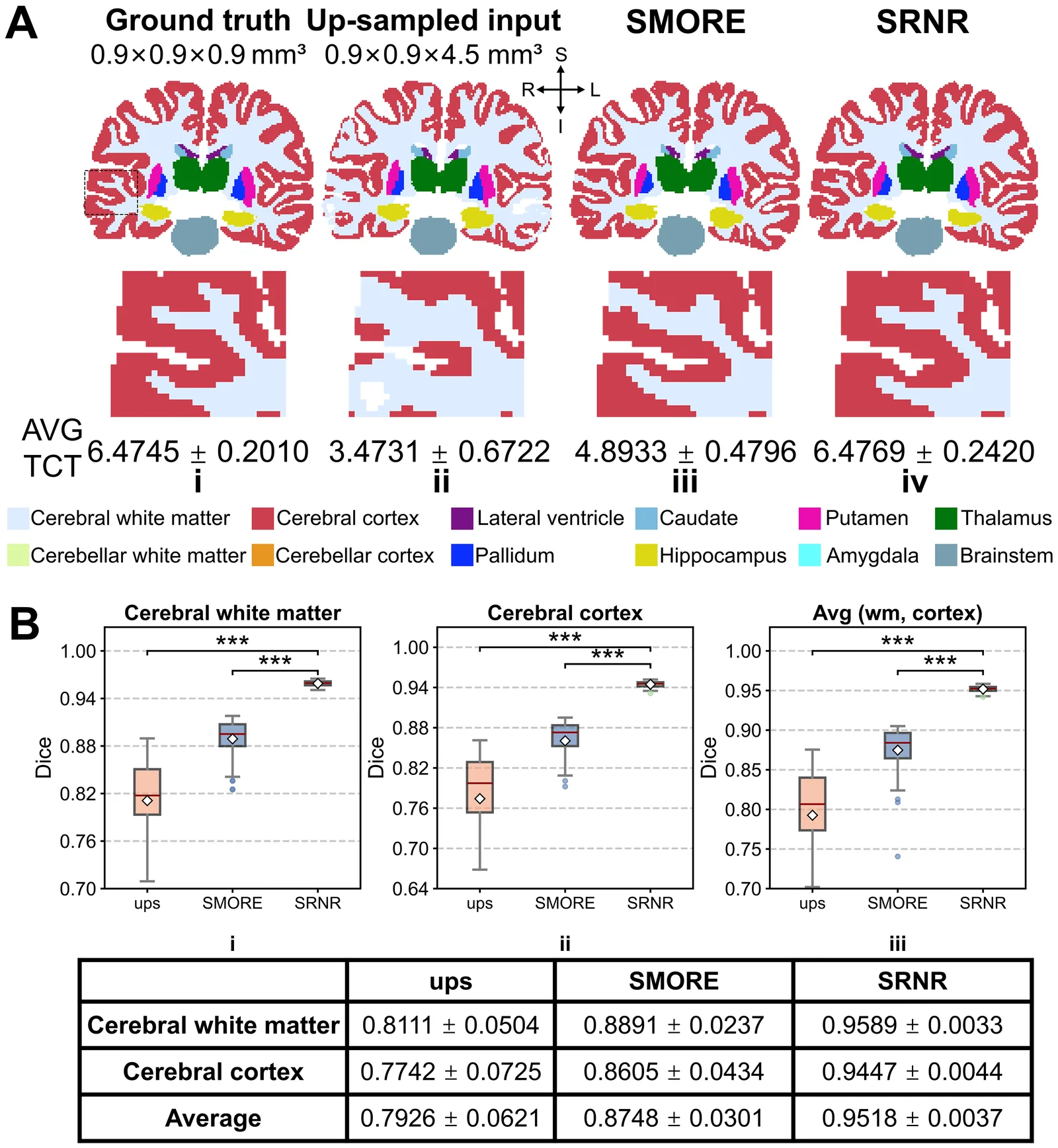
Comparison of volumetric segmentation accuracy between SRNR and SMORE in simulation experiments (0.4σ). (A) Exemplar coronal slices from a representative subject in the independent test set illustrate segmented brain structures derived from the high-resolution ground-truth image volume (i), the up-sampled thick-slice input (ii), and outputs generated by SMORE (iii) and SRNR (iv). The region of interest encompassing cortical gray and white matter, indicated by the black box, is shown in enlarged views. Group means ± standard deviations of the tissue contrast *t*-score (TCT) are provided for each method. (B) Box plots show Dice similarity coefficients for cerebral white matter (i), cerebral cortex (ii), and their average (iii). Results are presented for segmentations derived from the up-sampled inputs (ups) and outputs generated by SMORE and SRNR. White diamonds indicate the means, horizontal lines within the boxes indicate the medians, and box boundaries indicate the first and third quartiles. Whiskers extend to the most extreme observations within 1.5 times the interquartile range, and observations beyond the whiskers are shown individually. SRNR significantly outperformed up-sampled and SMORE in each metric (^***^P < 0.001, two-sided paired *t*-test, *n* = 59; one subject was excluded due to cortical reconstruction failure in the up-sampled volume). Corresponding group means ± standard deviations are listed in the accompanying table.

### 3.3 Empirical results

On empirical pediatric data, SRNR trained using noisy high-resolution Wave-MPRAGE reference volumes consistently produced high-quality super-resolved outputs compared with up-sampled input volumes across both acceleration settings and for all four subjects in the independent test set. For data acquired with an acceleration factor of R = 3 × 3, SRNR reduced the mean MAE from 0.049 to 0.028, increased the mean PSNR from 24.232 to 27.956 dB, and improved the mean SSIM from 0.886 to 0.926 (Fig. 10A, C). Comparable improvements were observed for data acquired at R = 3 × 4, with the mean MAE reduced to 0.030, PSNR increased to 27.571 dB, and SSIM improved to 0.911 (Fig. 10B, D). Qualitatively, the super-resolved outputs recovered fine anatomical details while effectively suppressing noise present in the accelerated acquisitions, resulting in enhanced tissue contrast and more faithful depiction of fine structural features.

**Fig. 10.**
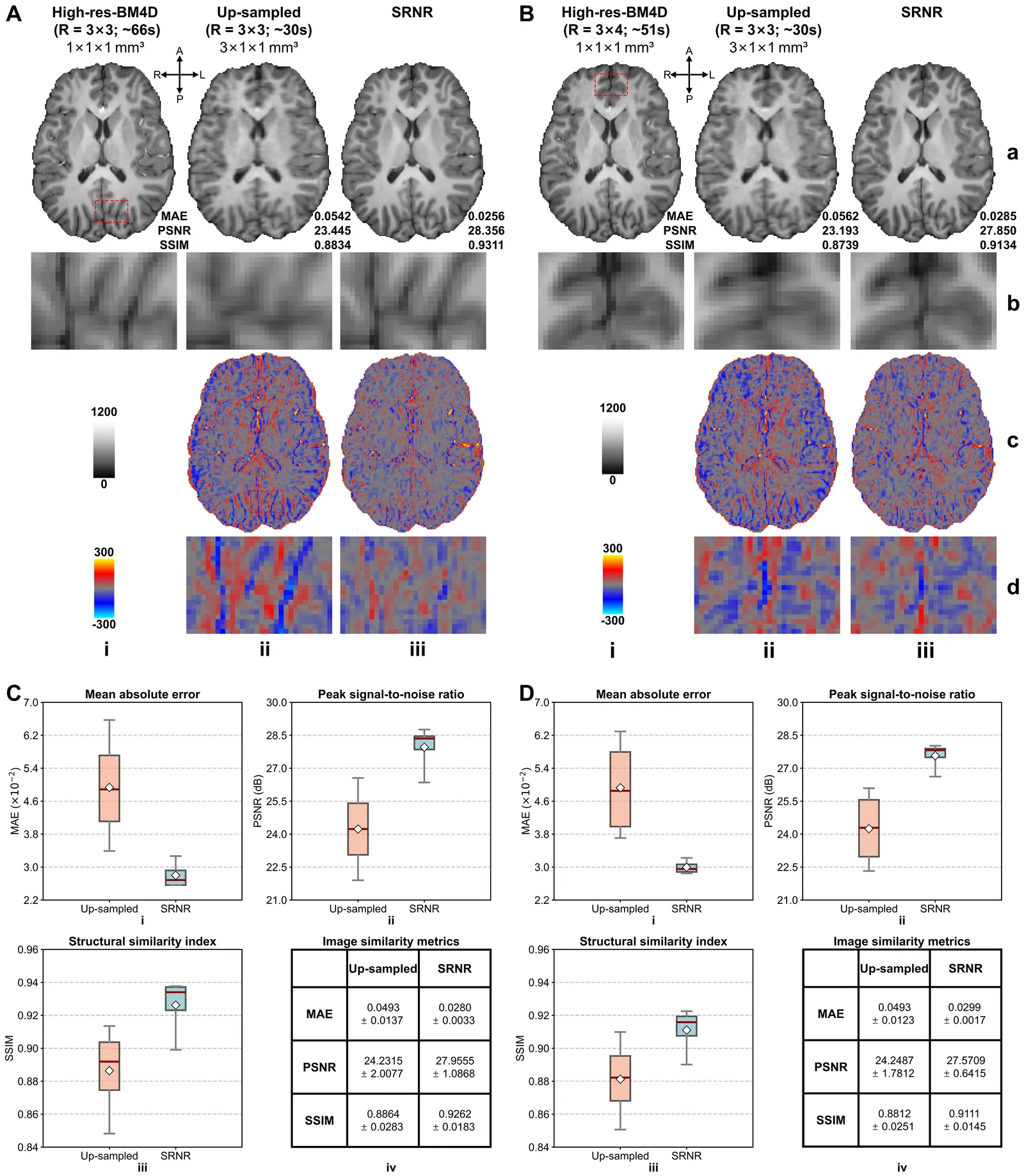
Image reconstruction quality in empirical experiments. Results are shown for data of a representative pediatric subject acquired with acceleration factors of R = 3 × 3 (A, C) and R = 3 × 4 (B, D). (A, B) Exemplar axial slices from a representative pediatric clinical subject in the independent test set illustrate the acquired accelerated high-resolution image volume (a, i), the up-sampled sagittal thick-slice image volume (a, ii), and the super-resolved output (a, iii), along with enlarged views (b). Difference maps depict the similarity of the up-sampled and super-resolved image volumes relative to the accelerated high-resolution image volume (c, d). Whole-brain-averaged MAE, PSNR, and SSIM for the exemplar subject are listed to quantitatively assess image similarity. (C, D) Box plots illustrate the whole-brain-averaged MAE (i), PSNR (ii), and SSIM (iii) across four evaluation subjects, computed for up-sampled thick-slice image volumes (up-sampled) and super-resolved outputs, relative to the accelerated high-resolution reference image volumes. The midline indicates the median, diamond markers denote the mean values, box edges denote the first and third quartiles, and whiskers represent the minimum and maximum values. The corresponding quantitative values are listed (iv).

Consistent gains were also observed in volumetric segmentation performance. At R = 3 × 3, Dice similarity coefficients derived from super-resolved predictions consistently exceeded those from the up-sampled image volumes (cerebral WM Dice: 0.939 vs. 0.913; cerebral cortex Dice: 0.889 vs. 0.836; average Dice across both tissues: 0.914 vs. 0.874; Fig. 11A). A similar trend was observed at R = 3 × 4 (cerebral WM Dice: 0.931 vs. 0.909; cerebral cortex Dice: 0.873 vs. 0.828; average Dice across both tissues: 0.902 vs. 0.868; Fig. 11B). In enlarged views, SRNR reconstructions visually corrected segmentation boundaries that were clearly erroneous in the up-sampled results. Dice similarity coefficients for all 12 individual brain structures, as well as for all structures combined, are reported in Supplementary Table S2.

**Fig. 11.**
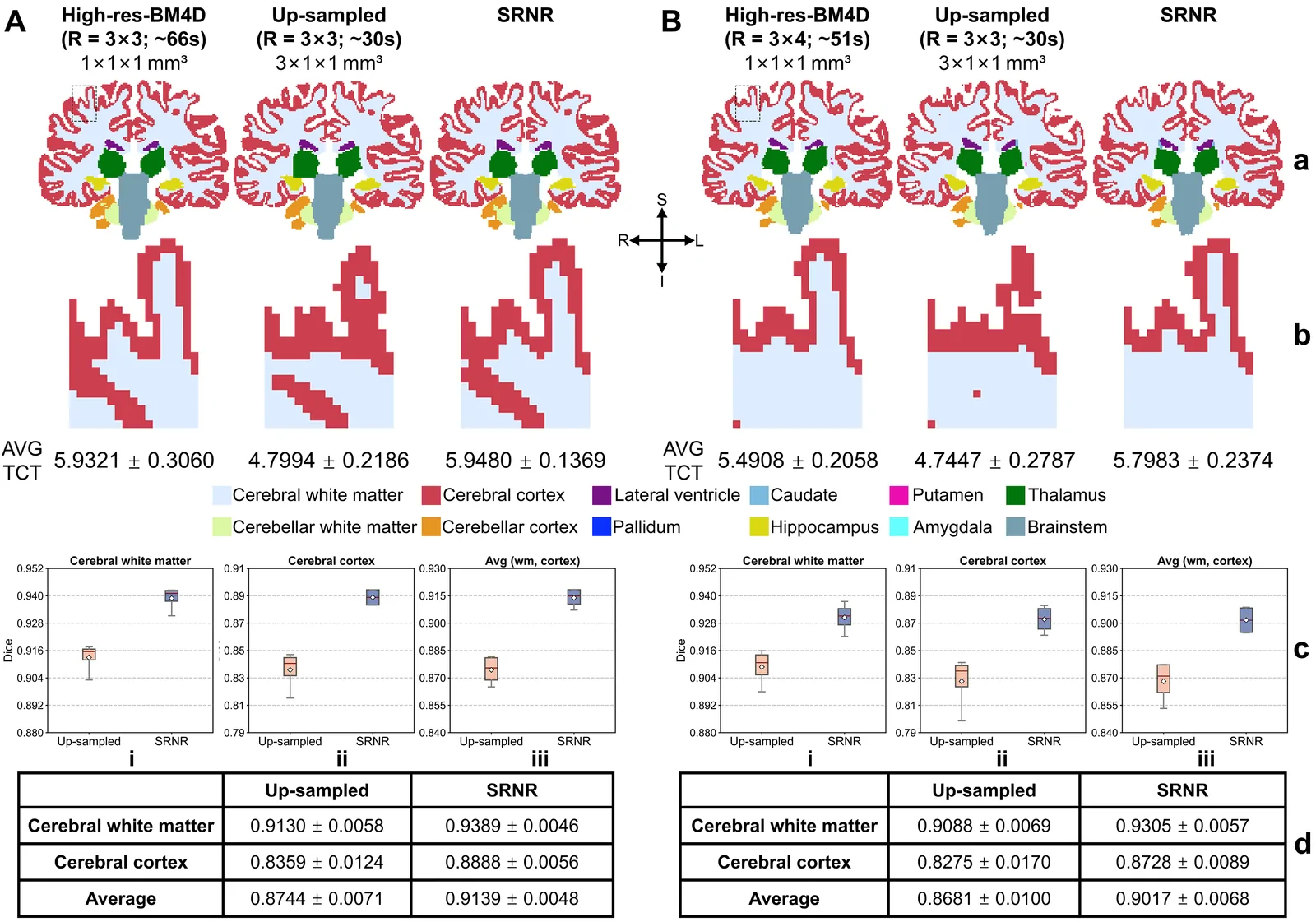
Volumetric segmentation in empirical experiments. Results are shown for data of a representative pediatric subject acquired with acceleration factors of R = 3 × 3 (A) and R = 3 × 4 (B). Exemplar coronal slices from a representative pediatric clinical subject show segmented brain structures derived from the high-SNR Wave-MPRAGE reference image volume (i), up-sampled thick-slice image volume (ii), and super-resolved output (iii), with enlarged views and average TCT values provided for comparison (b). Box plots illustrate the Dice similarity coefficients across four evaluation subjects, computed for segmentations derived from up-sampled thick-slice image volumes and super-resolved outputs for the cerebral white matter (i), cerebral cortex (ii), and their average (iii). The midline indicates the median, box edges indicate the first and third quartiles, and whiskers denote the minimum and the maximum values. Dice similarity coefficients for all 12 individual brain structures and for all structures combined are reported in Supplementary Table S2.

Super-resolved predictions also demonstrated superior tissue contrast, as quantified by the TCT. At R = 3 × 3, TCT reached 5.948 for the super-resolved predictions, compared with 5.932 for the high-resolution reference and 4.799 for the up-sampled image volumes. At R = 3 × 4, super-resolved predictions achieved a TCT of 5.798, exceeding both the high-resolution reference (5.491) and the up-sampled image volumes (4.745). The higher TCT values relative to the high-resolution reference are likely attributable to the denoising capability of SRNR.

SRNR further improved cortical surface reconstruction in the empirical dataset (Fig. 12). At an acceleration factor of R = 3 × 3, super-resolved predictions consistently yielded lower errors than the up-sampled inputs across all evaluated metrics, including whole-brain-averaged cortical thickness estimation (0.371 vs. 0.434), gray–white matter boundary positioning (0.363 vs. 0.496), and gray–CSF boundary positioning (0.507 vs. 0.624). Similar improvements were observed at R = 3 × 4, where super-resolved predictions achieved lower errors in cortical thickness estimation (0.405 vs. 0.501), gray–white matter boundary positioning (0.396 vs. 0.510), and gray–CSF boundary positioning (0.551 vs. 0.622), indicating more faithful recovery of cortical geometry under increased acceleration.

**Fig. 12.**
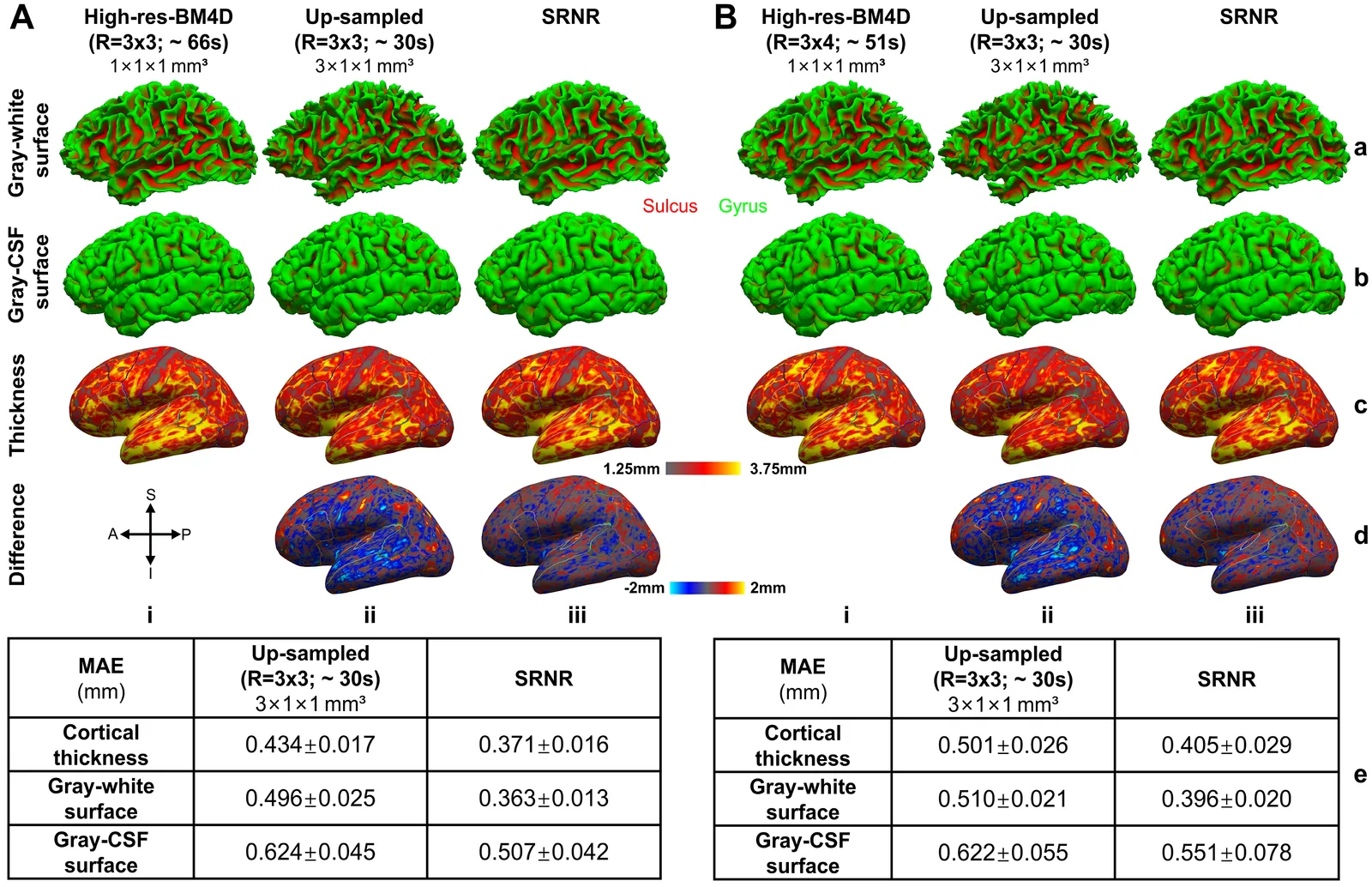
Cortical surface reconstruction and cortical thickness accuracy in empirical experiments. Results are shown for data of a representative pediatric subject acquired with acceleration factors of R = 3 × 3 (A) and R = 3 × 4 (B). The gray–white matter interface surfaces (a) and gray–CSF interface surfaces (b) reconstructed from the high-SNR Wave-MPRAGE reference image volume (i), up-sampled image volume (ii), and super-resolved output (iii) are shown. Left-hemispheric vertex-wise cortical thickness (c) and its difference relative to the high-resolution reference (d) are displayed on the inflated surface. Different cortical regions defined by the FreeSurfer cortical parcellation (i.e., aparc.annot) are depicted as colored outlines. Group means (± standard deviations) of the whole-brain-averaged absolute differences in the cortical thickness estimation and the displacements of the gray–white and gray–CSF surfaces estimated from the up-sampled and super-resolved predictions relative to the reference are quantified (e).

Evaluation results computed using the original, non-denoised scans are reported in Supplementary Note S2, Supplementary Table S2 and Supplementary Figures S6–S8, which also support the superior image quality and downstream morphometric performance of SRNR outputs.

### 3.4 Transfer learning results

SRNR demonstrated robust SR performance under transfer learning, even when only a limited number of pediatric clinical volumes were available for training. Specifically, the network was first pre-trained on the simulated pediatric dataset and subsequently fine-tuned using pediatric clinical data from only three subjects. For an acceleration factor of R = 3 × 3, evaluation on four held-out test subjects showed that direct application of the pre-trained network already led to effective improvements in image quality metrics. Fine-tuning further enhanced performance, achieving image quality comparable with that obtained by training the network from scratch using data from eight subjects (Fig. 13). Quantitative image quality metrics were consistent with these observations. The fine-tuned SRNR achieved a mean MAE of 0.031, PSNR of 27.248 dB, and SSIM of 0.913, compared with 0.028, 27.956 dB, and 0.926, respectively, for models trained from scratch. Importantly, we also evaluated transfer learning using the HCP adult dataset for pre-training followed by fine tuning on pediatric clinical data. The results showed that regardless of whether pediatric or adult datasets were used for pre-training, fine-tuned models achieved comparable performance with those trained from scratch (Supplementary Note S3 and Supplementary Fig. S9). This cross-population transferability indicates that SRNR can learn transferable representations for resolution enhancement that are robust to differences in brain anatomy and developmental stage. These results indicate that fine tuning with very limited clinical data can effectively reduce the domain gap between simulated and empirical data while maintaining high SR performance. Fine-tuning results for R = 3 × 4 are presented in Supplementary Figures S10–S11, which show similar improvements.

**Fig. 13.**
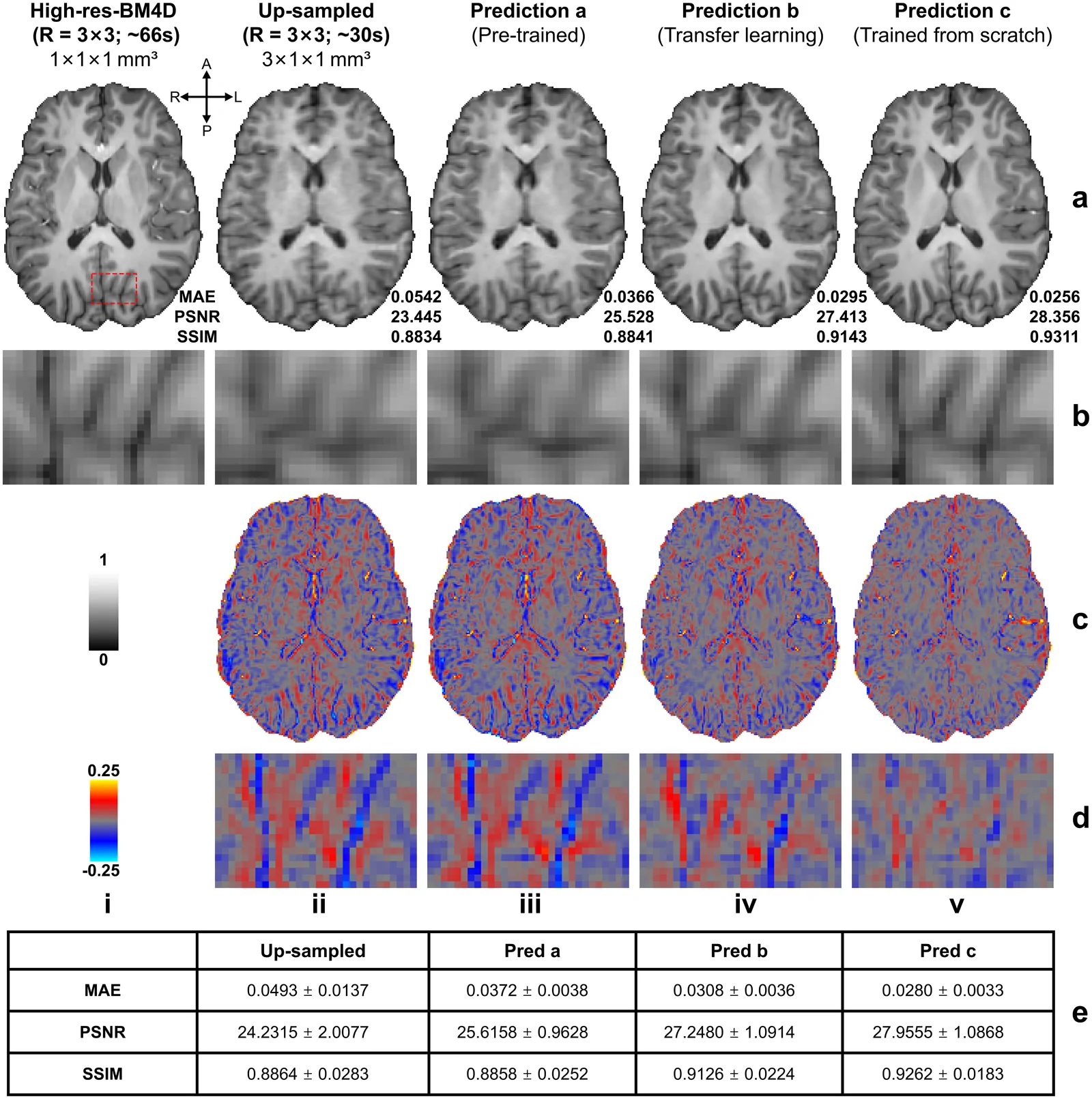
Transfer learning results on empirical data. Exemplar axial slices from a representative pediatric clinical subject in the independent test set (R = 3 × 3) are shown for the acquired accelerated high-resolution reference image volume (a, i), the up-sampled sagittal thick-slice image volume (a, ii), the super-resolved prediction obtained by applying the pre-trained network trained on the Calgary Preschool MRI Dataset (a, iii), the fine-tuned network using three pediatric clinical cases (a, iv), and the network trained from scratch using eight pediatric clinical cases (a, v). Difference maps depict the similarity of the up-sampled and super-resolved image volumes relative to the accelerated high-resolution reference image volume (c, d). Enlarged views of the region of interest are shown in (b) and (d). Whole-brain-averaged MAE, PSNR, and SSIM for the exemplar subject are listed to quantify image similarity. The whole-brain-averaged MAE, PSNR, and SSIM across four evaluation subjects, computed for up-sampled thick-slice image volumes (up-sampled) and super-resolved outputs relative to the accelerated high-resolution reference image volumes are listed (e).

## 4 Discussion

This study demonstrates that high-quality SR can be achieved without high-SNR training targets by leveraging the statistical properties of noise inherent to accelerated MRI acquisitions. The proposed SRNR paradigm enables training using noisy high-resolution reference image data acquired under clinically feasible conditions, yet consistently generates super-resolved outputs with high structural fidelity and reduced noise. Across both simulated and pediatric clinical datasets, SRNR exhibited strong agreement with high-resolution references and reliably preserved anatomically meaningful features relevant to downstream analyses, including tissue segmentation and cortical surface reconstruction.

SRNR operates under the assumption that noise in the high-resolution reference volumes is zero mean and statistically independent of the low-resolution inputs, consistent with the assumption underlying the Noise2Noise framework. The validity of this assumption was examined in simulation experiments, in which controlled zero-mean Gaussian noise was added to noise-free high-resolution reference volumes. Accurate SR reconstructions were obtained even at elevated noise levels, supporting the theoretical rationale of the method. In empirical MRI magnitude data, noise is more accurately modeled by a Rician distribution, and under typical SNR conditions encountered in clinical MRI, Rician noise can be reasonably approximated as zero-mean Gaussian. However, accelerated clinical MRI may deviate from this idealized noise model. Spatially varying noise amplification associated with coil sensitivity and parallel imaging, reconstruction-related intensity bias, motion, and other structured artifacts may give rise to non-zero-mean or input-dependent errors that violate the assumptions of SRNR. Unlike independent zero-mean noise, such errors may not cancel in the expected training objective and may instead be incorporated into the learned mapping, potentially biasing the reconstruction or compromising regional anatomical fidelity. The empirical findings, therefore, support the feasibility of SRNR under the acquisition conditions studied here and do not establish robustness to the full range of noise and artifact conditions encountered in clinical practice.

The primary contribution of SRNR lies in eliminating the requirement for high-resolution, high-SNR reference data for training SR models. This offers two key advantages. First, it substantially improves the feasibility of acquiring training data in prospective studies. Second, it enables effective model training on retrospective datasets in which high-SNR reference data are unavailable. These advantages are particularly important given the inherent trade-off between spatial resolution and SNR in MRI. By exploiting complementary information from high-resolution, low-SNR references and low-resolution, high-SNR inputs, SRNR enables more efficient utilization of clinically realistic acquisitions. The evaluations presented in this study provide preliminary evidence of the practical utility of SRNR, extending beyond conventional image quality metrics to downstream analyses that are critical to assessing its potential clinical use.

The fine-tuning experiments further underscore the practical value of SRNR in scenarios where acquiring high-resolution clinical reference volumes is especially challenging. During pre-training on the simulated dataset, SRNR learned effective representations for recovering high-resolution anatomical structures, which were subsequently refined through fine tuning using a small number of pediatric clinical data. Despite differences in subject populations and acquisition protocols between the simulated data and the pediatric clinical MRI, the learned representations generalized well and adapted effectively during fine tuning. This capability to leverage large-scale public datasets and subsequently adapt to data-limited clinical settings substantially lowers the barrier to deploying SR methods in routine medical imaging practice.

From the perspective of clinical translation, SRNR simplifies both model development and potential clinical integration. During model development, the ability to train using routinely acquired noisy high-resolution images substantially reduces the burden of collecting specialized high-SNR reference datasets, making it practical to develop or update SR models using data generated during routine clinical practice. As demonstrated by the fine-tuning experiments, models pretrained on large public datasets can also be efficiently adapted to institution-specific imaging protocols using only a limited amount of local data. During deployment, SRNR does not require modifications to the inference procedure beyond those of the underlying SR network, since it modifies only the training paradigm. Consequently, the trained model can be incorporated as a standard post-processing step following routine MRI reconstruction without requiring changes to existing acquisition protocols or scanner hardware, thereby facilitating integration into clinical workflows.

Several limitations should be acknowledged. First, BM4D-denoised high-resolution volumes were used as surrogate high-SNR references for quantitative evaluation, despite not representing truly noise-free ground truth. This approach was motivated by both the impracticality of repeated high-resolution acquisitions in routine pediatric imaging and previous studies demonstrating that BM4D denoising can achieve image quality comparable with that obtained by averaging multiple repeated scans (Tian, Zaretskaya, et al., 2021). Therefore, the reported quantitative metrics should be interpreted as relative measures against a high-SNR surrogate reference rather than absolute measures of reconstruction fidelity. Although BM4D improves image SNR, residual noise and potential smoothing of fine anatomical structures may introduce unknown evaluation bias, which could either overestimate or underestimate reconstruction performance. Second, the simulation targets were generated by nonlinearly registering an atlas to individual Calgary Preschool MRI scans. Although this approach provides controlled, anatomically plausible, and noise-free surrogate targets, atlas registration may not fully preserve subject-specific fine anatomy, cortical folding patterns, or subtle local morphological variation. Accordingly, simulation-based image and morphometric metrics should be interpreted as evidence that SRNR can learn from noisy targets under controlled conditions and not as definitive evidence of subject-specific anatomical or morphometric fidelity. Third, the empirical cohort comprised 12 healthy pediatric participants acquired on a single scanner using a single protocol, with eight subjects used for training and validation and only four held out for testing. The clinical findings should, therefore, be interpreted as preliminary feasibility evidence and not as definitive validation of broad clinical generalizability across sites, scanners, age ranges, acquisition protocols, or disease states. Finally, the comparative evaluation included SMORE as the only external SR baseline; its scan-specific self-supervised formulation differs fundamentally from SRNR’s externally supervised training setting. The comparison should, therefore, be interpreted as evidence of performance within the evaluated protocol, rather than as a universal ranking relative to the broader range of supervised, self-supervised, and generative SR methods. In addition, although SRNR is designed as a general training paradigm, its empirical evaluation in this study was limited to two closely related U-Net architectures. Its generality across materially different SR network architectures remains to be established.

Future work should evaluate SRNR in larger, multicenter, and more diverse pediatric cohorts spanning different scanners, acquisition protocols, age ranges, and clinical conditions to better assess clinical generalizability. Further studies should also assess SRNR under clinically realistic non-ideal conditions, and benchmark it against a broader range of supervised and self-supervised SR methods across diverse network architectures. Beyond pediatric neuroimaging, SRNR may also be applicable to other clinical imaging scenarios in which high-resolution, high-SNR reference volumes are difficult to obtain. These include low-field MRI, pathological conditions, as well as other anatomical regions including cardiac, abdominal, spinal, and musculoskeletal imaging, where subject motion, time constraints, and SNR limitations remain major challenges. More broadly, the underlying principles of SRNR may inspire related developments in other imaging modalities, such as CT and ultrasonography, in which image quality is similarly constrained by noise and limited spatial resolution.

## 5 Conclusion

This study introduces the SRNR training paradigm and demonstrates its effectiveness through validation on both simulated and real clinical datasets. By relaxing the requirement for high-SNR, high-quality training targets, SRNR substantially reduces the data acquisition burden typically associated with supervised medical image SR. This capability may facilitate model development and improve the practicality of deploying deep learning-based SR methods in clinical practice. Importantly, SRNR enables the effective use of accelerated and low-resolution MRI inputs, providing a promising pathway toward reducing scan times in pediatric imaging and potentially decreasing the need for sedation. Beyond pediatric MRI, SRNR may be extended to a wide range of SR applications and imaging scenarios.

## Supplementary Material

Supplementary Material

## Data Availability

The pediatric clinical data from the West China Second University Hospital are protected due to patient privacy. Some data can be made available for academic purposes from the corresponding author upon reasonable request and with authorization from the hospital. T1w MRI data from Calgary Preschool MRI Dataset are publicly available (https://portal.conp.ca/dataset?id=projects/Calgary-Preschool-MRI-Dataset). T1w MRI data from the Human Connectome Project, WU-Minn-Ox Consortium, are publicly available (https://www.humanconnectome.org). The source code of BM4D is publicly available (https://webpages.tuni.fi/foi/GCF-BM3D/). The source code and weights of SRNR are publicly available (https://github.com/birthlab/SRNR).
