# Supplementary material for: SRNR: Deep learning-based MRI super-resolution using noisy high-resolution reference data
Source: Imaging Neurosci (Camb). 2026 Nov 3;4:IMAG.a.1433. doi: 10.1162/IMAG.a.1433 (PMC13732491; doi:10.1162/IMAG.a.1433)
Supplement: Supplementary file 1 [file IMAG.a.1433_supp.pdf]

SRNR: Deep learning-based MRI **S**uper-**R**esolution using **N**oisy high-resolution **R**eference data

Muye Zhang[1#], Hongjia Yang[1#], Zihan Li[1#], Jiaxin Xiao[1], Wanqing Liu[2], Yuhang He[1], Ting Yin[3], Jialan Zheng[1], Mingxuan Liu[1], Wei Liu[4], Congyu Liao[5], Berkin Bilgic[6,7,8], Jonathan R. Polimeni[6,7,8], Susie Y. Huang[6,7,8], Yi Liao[2], Ziyu Li[9*], Haibo Qu[2*], Qiyuan Tian[1*]

[1] School of Biomedical Engineering, Tsinghua University, Beijing, China;
[2] Department of Radiology, West China Second University Hospital, Sichuan University, Chengdu, China;
[3] MR Research Collaborations, Siemens Healthineers Ltd., Chengdu, China;
[4] Siemens Healthineers AG, Erlangen, Germany;
[5] Department of Radiology & Biomedical Imaging, University of California San Francisco, San Francisco, CA, United States;
[6] Athinoula A. Martinos Center for Biomedical Imaging, Department of Radiology, Massachusetts General Hospital, Charlestown, MA, United States;
[7] Department of Radiology, Harvard Medical School, Boston, MA, United States;
[8] Harvard-MIT Program in Health Sciences and Technology, Massachusetts Institute of Technology, Cambridge, MA, United States;
[9] Oxford Centre for Integrative Neuroimaging, FMRIB, Nuffield Department of Clinical Neurosciences, University of Oxford, Oxford, United Kingdom.

[#] Muye Zhang, Hongjia Yang, and Zihan Li contributed equally to this work.
[*]Correspondence to: Qiyuan Tian, Ph.D., Center for Biomedical Imaging Research at Tsinghua University, 30 Shuangqing Road, Haidian District, Beijing, China, 100084. E-mail: qiyuantian@tsinghua.edu.cn. Haibo Qu, M.D., No. 20, Section 3, Renmin South Road, Chengdu, Sichuan Province, China, 610041. E-mail: windowsqhb@126.com. Ziyu Li, Ph.D., Oxford Centre for Integrative Neuroimaging, FMRIB, John Radcliffe Hospital, Oxford, United Kingdom, OX3 9DU. E-mail: ziyu.li@ndcn.ox.ac.uk.

Supplementary Note 1: Simulation experiments using the HCP dataset

Method

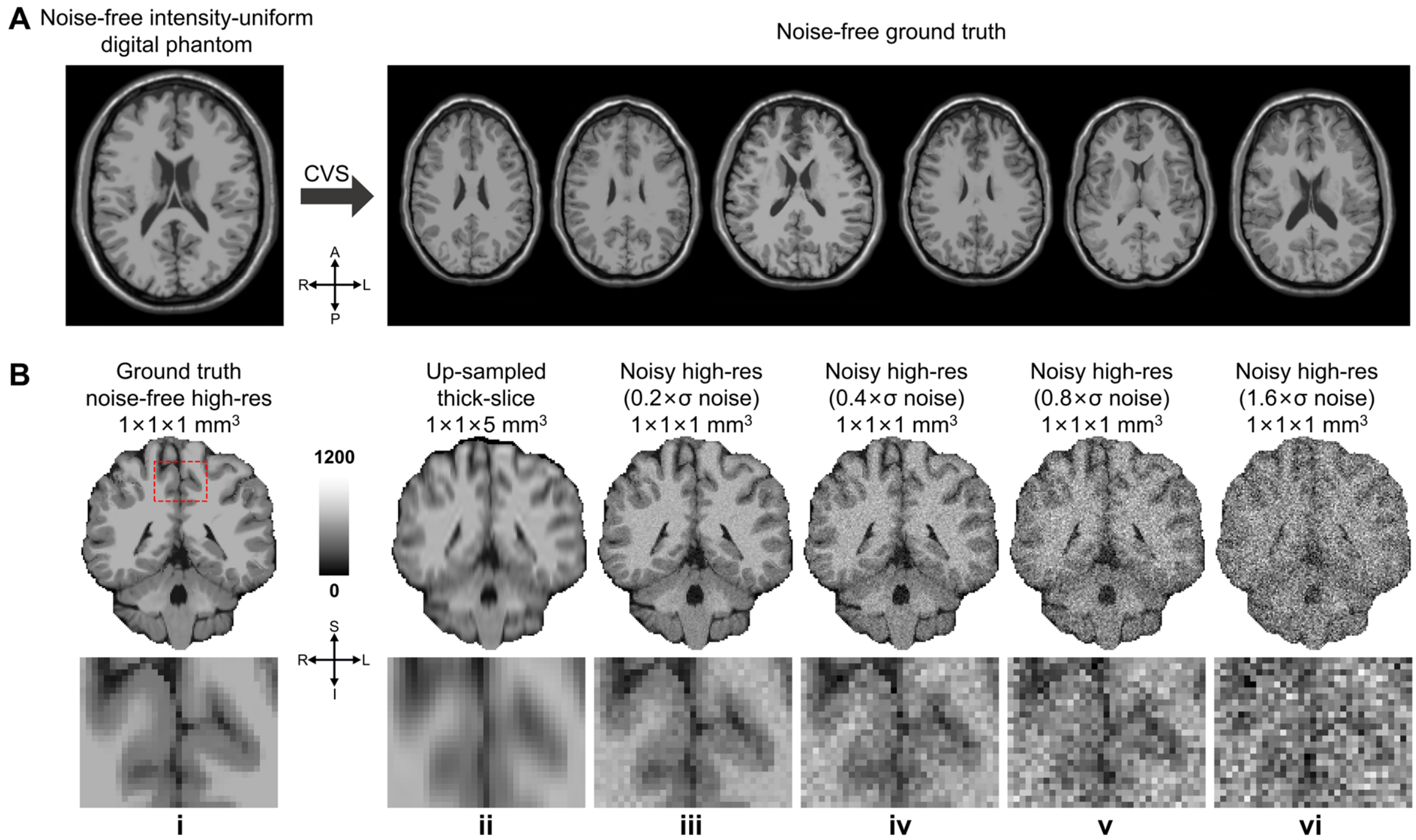

Fig. S1. Generation of simulated data. A. A noise-free, intensity-uniform T1w digital phantom was registered to T1w image volumes from different HCP subjects using FreeSurfer's CVS registration and served as the noise-free ground-truth reference. **B.** Exemplar coronal slices from a representative subject illustrate the noise-free high-resolution ground-truth image volume (i), the up-sampled low-resolution image volume (ii), and the simulated noisy high-resolution image volumes (iii–vi, generated by adding Gaussian noise with standard deviations of 0.2 (iii), 0.4 (iv), 0.8 (v), and 1.6 (vi) times the standard deviation of voxel intensities within the brain mask). Enlarged views of a region of interest (red box) encompassing cortical gray and white matter are shown for clarity.

To demonstrate the efficacy of SRNR and to evaluate its performance across different noise levels, we first conducted experiments using simulated data. To obtain ground-truth volumes free of the thermal noise inherent in MRI, we employed a noise-free, intensity-uniform T1w digital phantom (1×1×1 mm³) from BrainWeb (Cocosco, C.A. et al., 1997). To increase the number of brain samples and capture a representative range of anatomical variability, this phantom was registered to high-resolution T1w image volumes

(1×1×1 mm³) from 90 healthy subjects in the Human Connectome Project (HCP) (Glasser et al., 2013, 2016), using the Combined Volumetric and Surface (CVS) registration algorithm implemented in FreeSurfer (Fischl, 2012). CVS integrates surface-based morphometry with volume-based refinement, preserving cortical folding patterns while achieving accurate alignment of subcortical structures (Postelnicu et al., 2009) (Fig. S1A). Noisy high-resolution reference image volumes were generated by adding zero-mean Gaussian noise ($\mu = 0$; $\sigma = 0.2, 0.4, 0.8, 1.6$ times the standard deviation of voxel intensities within brain masks) to the registered volumes (Fig. S1B). Corresponding low-resolution thick-slice image volumes with a spatial resolution of 1×1×5 mm³ were simulated by applying blurring followed by down-sampling along the superior-inferior axis, using a one-dimensional 48th-order Hamming-windowed low-pass filter. These low-resolution volumes were subsequently up-sampled to 1-mm isotropic resolution using cubic spline interpolation and served as the network inputs.

For the simulation experiments, 45 co-registered digital phantoms were used for training and validation, and an independent set of 45 phantoms was held out for testing. Overlapping 3D patches of 96 × 96 × 96 voxels were extracted using a deterministic sliding window covering the bounding box of the brain mask. Both the input and target volumes were Z-score normalized within the brain mask using the mean and standard deviation of the input volume. No spatial augmentation was applied. The network used 64 base feature channels and was optimized using Adam with a brain-mask-weighted mean squared error (Kingma & Ba, 2017). Training was performed for 50 epochs with a batch size of one. The learning rate was initialized at 1×10^{-3} and reduced to 3×10^{-4}

and 1×10^{-4} at epochs 15 and 35, respectively. Within the training and validation cohort, patches were divided into training and validation sets at a ratio of 80%/20%.

Result

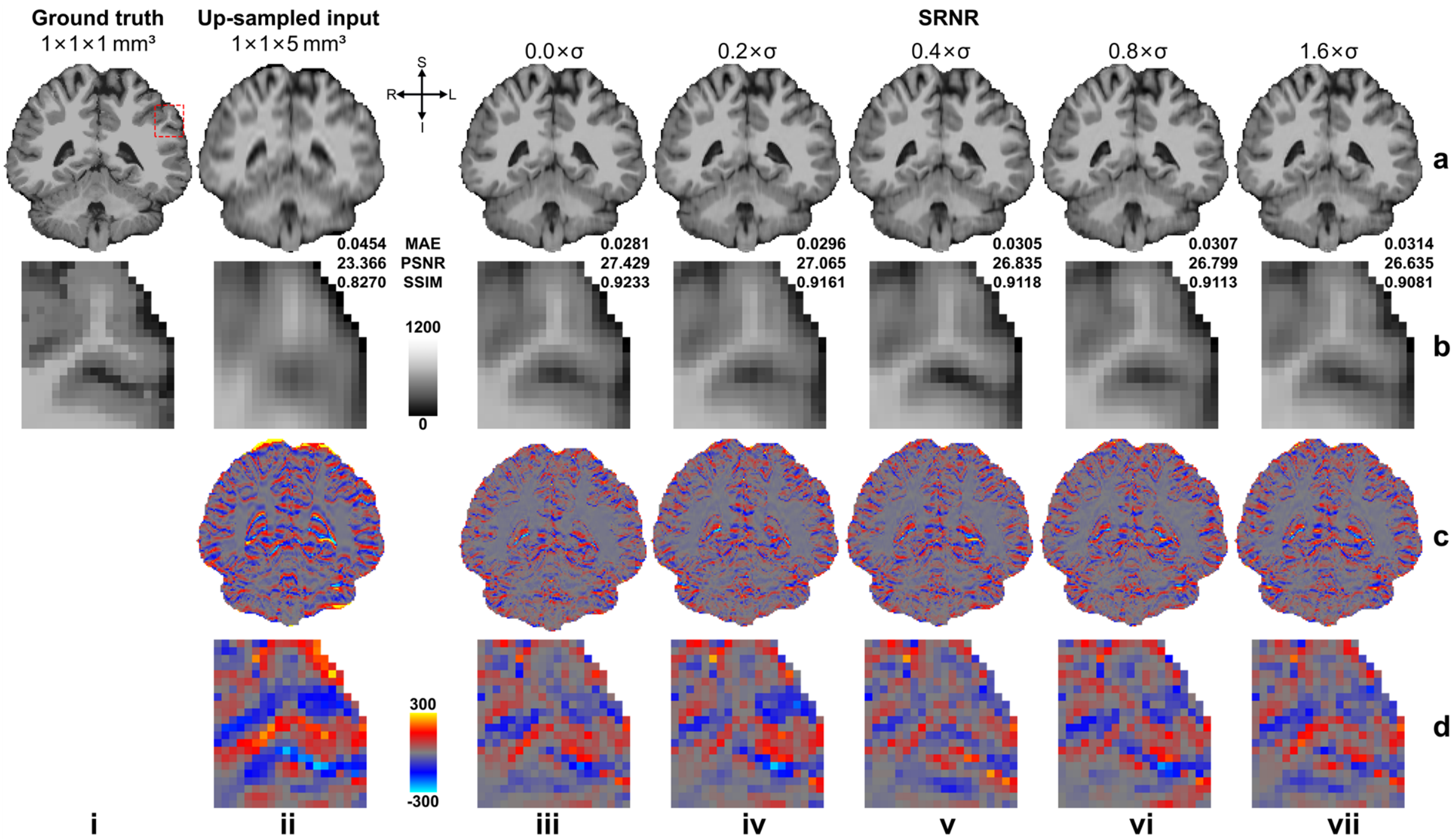

Fig. S2. Simulation experiments. Exemplar coronal slices from a representative subject in the independent test set illustrate the ground-truth high-resolution image volume (a, i), the up-sampled low-resolution input (a, ii), and the super-resolved prediction from neural networks trained using noise-free ground truth (a, iii), as well as SRNR super-resolved predictions from neural networks trained using simulated noisy high-resolution image volumes (a, iv–vii; with noise standard deviation equal to 0.2 (iv), 0.4 (v), 0.8 (vi), and 1.6 (vii) times the standard deviation of voxel intensities within the brain mask). Enlarged views of a region of interest (red box) are also shown for clarity (b). Difference maps (c, d) depict the similarity of the up-sampled and super-resolved image volumes relative to the ground truths. The whole-brain-averaged mean absolute error (MAE), peak signal-to-noise ratio (PSNR), and structural similarity index (SSIM) for the exemplar subject are listed to quantify image similarity.

Simulation experiments demonstrated that SRNR produced super-resolved image volumes that were visually highly consistent whether the networks were trained using high-SNR or noisy high-resolution ground truths (Fig. S2a). Compared with up-sampled low-resolution inputs, SRNR outputs exhibited markedly enhanced textural and structural detail and sharper tissue boundaries. Enlarged views further showed well-preserved gray-white matter contrast and spatial organization consistent with the ground-truth image

volumes (Fig. S2b). Importantly, increasing noise levels in the training references had no appreciable impact on the quality of the super-resolved outputs (Fig. S2a, iii-vii). Even when the noisiest high-resolution references were used for training, the resulting super-resolved image volumes remained visually indistinguishable from those obtained using noise-free ground truths (Fig. S2a, iii vs. Fig. S2a, vii). This observation is consistent with the theoretical expectation that SRNR converges to clean, high-resolution outputs even when trained with noisy reference data. Accordingly, residual maps between the super-resolved and ground-truth image volumes contained little structured anatomical information and exhibited substantially smaller errors than those observed for the up-sampled inputs (Fig. S2c, d).

Quantitative analysis further confirmed that SRNR significantly improved super-resolved image quality relative to up-sampled inputs and achieved strong agreement with the ground truths across all noise conditions (Fig. S3A, C). Specifically, SRNR reduced the mean MAE from 0.041 to 0.027–0.030 and increased the mean PSNR from 24.272 to 27.093–27.824 dB, as well as the mean SSIM from 0.843 to 0.912–0.925 (two-sided paired *t*-test, $P < 0.001$). As noise levels increased, image similarity between the noisy training references and the ground truths deteriorated markedly, following an approximately linear decline (Fig. S3B). In contrast, the similarity of SRNR super-resolved predictions exhibited only a modest reduction. Even under the highest noise condition ($1.6 \times \sigma$), performance degradation remained limited, with changes of only 1.1% in MAE, 0.731 dB in PSNR, and 1.4% in SSIM relative to the high-SNR setting. Moreover, super-resolved predictions obtained using noisy and noise-free training references were highly

consistent (MAE: 0.016–0.017; PSNR: 32.010–32.764dB; SSIM: 0.970–0.975; Fig. S3D), indicating that training with noisy reference volumes introduced negligible performance loss.

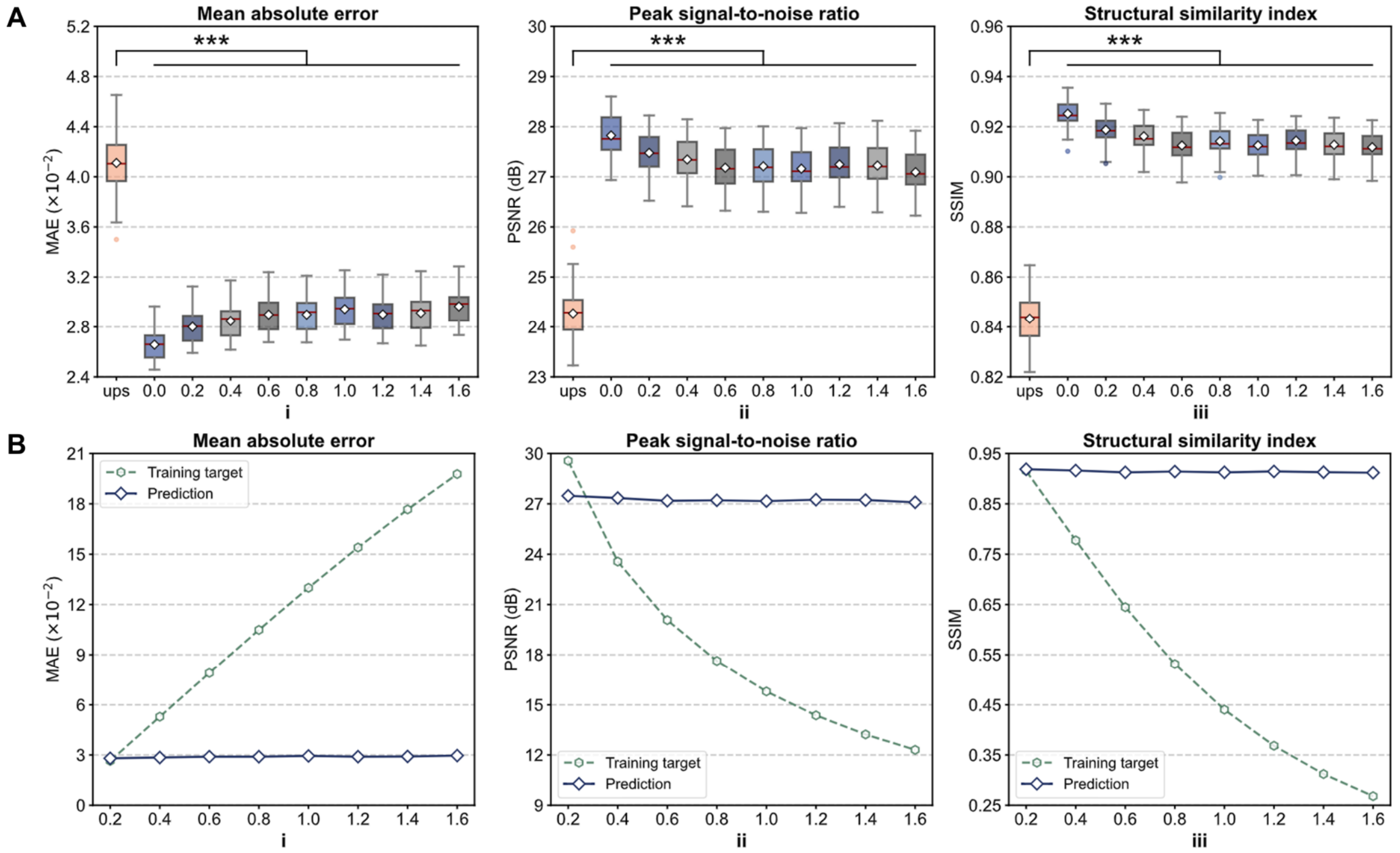

C

Image similarity metrics of SRNR predictions compared to ground truths

	Up-sampled	0.0×σ	0.2×σ	0.4×σ	0.6×σ	0.8×σ	1.0×σ	1.2×σ	1.4×σ	1.6×σ
MAE	0.0411 ± 0.0024	0.0266 ± 0.0013	0.0280 ± 0.0014	0.0285 ± 0.0014	0.0290 ± 0.0014	0.0290 ± 0.0014	0.0294 ± 0.0014	0.0290 ± 0.0014	0.0291 ± 0.0015	0.0296 ± 0.0014
PSNR	24.2721 ± 0.5554	27.8242 ± 0.3875	27.4773 ± 0.3806	27.3457 ± 0.3932	27.1812 ± 0.3897	27.2107 ± 0.3831	27.1622 ± 0.3752	27.2464 ± 0.3753	27.2246 ± 0.4003	27.0931 ± 0.3786
SSIM	0.8433 ± 0.0097	0.9252 ± 0.0055	0.9187 ± 0.0054	0.9162 ± 0.0057	0.9125 ± 0.0060	0.9142 ± 0.0057	0.9125 ± 0.0054	0.9144 ± 0.0055	0.9128 ± 0.0056	0.9118 ± 0.0057

D

Image similarity metrics of SRNR predictions compared to predictions of NN trained on noise-free ground truths

	0.2×σ	0.4×σ	0.6×σ	0.8×σ	1.0×σ	1.2×σ	1.4×σ	1.6×σ
MAE	0.0156 ± 0.0005	0.0161 ± 0.0006	0.0167 ± 0.0006	0.0170 ± 0.0005	0.0171 ± 0.0005	0.0168 ± 0.0005	0.0161 ± 0.0006	0.0173 ± 0.0005
PSNR	32.7637 ± 0.2671	32.5346 ± 0.3016	32.1453 ± 0.2817	32.0811 ± 0.2581	32.1574 ± 0.2541	32.2497 ± 0.2613	32.5974 ± 0.2965	32.0096 ± 0.2678
SSIM	0.9749 ± 0.0012	0.9727 ± 0.0013	0.9704 ± 0.0014	0.9702 ± 0.0014	0.9706 ± 0.0011	0.9711 ± 0.0013	0.9731 ± 0.0013	0.9696 ± 0.0015

Fig. S3. Image similarity metrics of simulation experiments. A. Box plots illustrate the group mean (± standard deviation) of whole-brain-averaged MAE (i), PSNR (ii), and SSIM (iii) across 45 evaluation subjects. Metrics were computed for the up-sampled low-resolution image volumes (ups) and for super-resolved outputs from neural networks trained on noise-free ground truths (i.e., 0.0×σ) and on simulated noisy high-resolution image volumes (with noise standard deviations ranging from 0.2 to 1.6 times the standard deviation of voxel intensities within the brain mask), all reported relative to the noise-free

high-resolution ground truths. The midline indicates the median, box edges indicate the first and third quartiles. Whiskers extend to the most extreme observations within 1.5 times the interquartile range, and observations beyond the whiskers are shown individually. Statistical significance for comparisons between up-sampled and super-resolved outputs is indicated by asterisks ($^{***}P$ < 0.001, two-sided paired *t*-test, *n* = 45). **B.** Line plots show the group mean MAE (i), PSNR (ii), and SSIM (iii) of the simulated noisy training references (green dashed lines) and the corresponding super-resolved outputs (blue solid lines) across varying noise levels, both calculated relative to the ground truths. **C**. The group mean (± standard deviation) of the quantitative values shown in A is listed. **D.** The group mean (± standard deviation) of whole-brain-averaged MAE, PSNR, and SSIM for super-resolved outputs from neural networks trained with noisy high-resolution image volumes, reported relative to those from neural networks trained with noise-free ground truths, across 45 evaluation subjects, is listed.

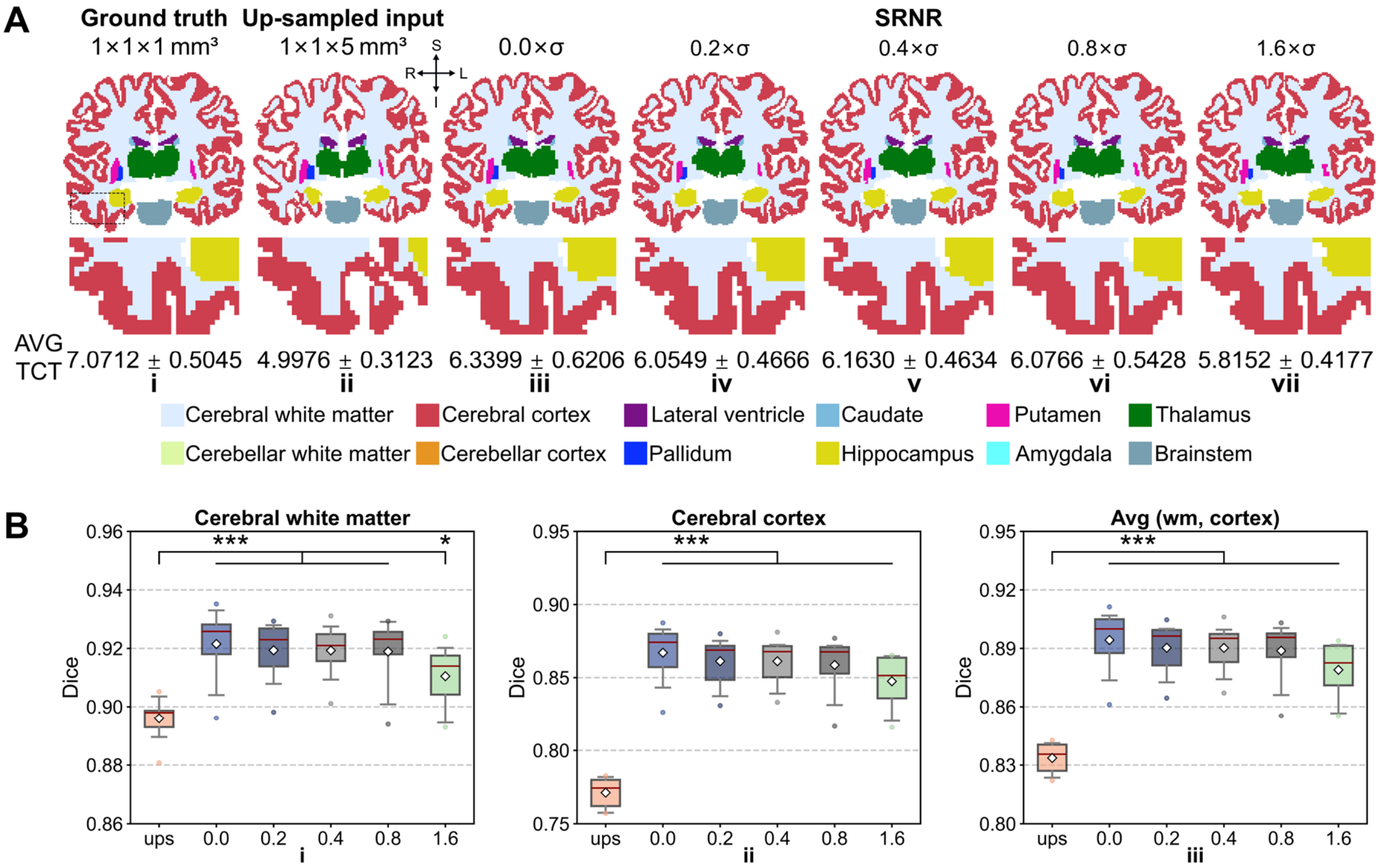

	ups	0.0×σ	0.2×σ	0.4×σ	0.8×σ	1.6×σ
**Cerebral white matter**	0.8960 ± 0.0067	0.9215 ± 0.0119	0.9194 ± 0.0097	0.9193 ± 0.0085	0.9189 ± 0.0114	0.9105 ± 0.0102
**Cerebral cortex**	0.7712 ± 0.0097	0.8670 ± 0.0188	0.8613 ± 0.0165	0.8612 ± 0.0152	0.8587 ± 0.0190	0.8475 ± 0.0178
**Average**	0.8336 ± 0.0075	0.8943 ± 0.0153	0.8903 ± 0.0130	0.8903 ± 0.0117	0.8888 ± 0.0152	0.8790 ± 0.0138

Fig. S4. Volumetric segmentation of simulation experiments. A. Exemplar coronal slices from a representative subject in the independent test set illustrate segmented brain structures derived from the high-resolution, noise-free ground-truth image volume (i), the up-sampled thick-slice image volume (ii), and super-resolved outputs from neural networks trained on high-resolution reference data with varying noise levels (iii-vii). Enlarged views of a region of interest (black box) are also shown for clarity. Average

Tissue Contrast t-score (TCT) values are listed. **B.** Box plots illustrate the group mean (± standard deviation) Dice similarity coefficients across 45 evaluation subjects, computed for segmentations derived from up-sampled thick-slice image volumes and from SRNR super-resolved outputs for cerebral white matter (i), cerebral cortex (ii), and their average (iii). The midline indicates the median, box edges indicate the first and third quartiles. Whiskers extend to the most extreme observations within 1.5 times the interquartile range, and observations beyond the whiskers are shown individually. Outliers beyond the whiskers are shown as individual points. Statistical significance for comparisons between up-sampled (ups) and super-resolved outputs is indicated by asterisks ($^{*}P < 0.05$, $^{***}P < 0.001$, two-sided paired *t*-test, $n = 45$). Dice similarity coefficients for all 12 individual brain structures and for all structures combined are reported in Table S3.

Volumetric segmentation of cerebral WM and cerebral cortex derived from SRNR-reconstructed simulated data exhibited high accuracy. Across training references with varying noise levels, SRNR consistently produced segmentation results that were visually comparable to the ground truths and demonstrated finer anatomical detail than those obtained from up-sampled image volumes (Fig. S4A). In localized regions, SRNR reconstructions corrected structural errors observed in the up-sampled segmentations. Quantitatively, perceptual tissue contrast in the super-resolved predictions, as measured by the TCT, was substantially improved (5.815–6.340) relative to the up-sampled image volumes (4.998) and approached that of the ground truths (7.071). Dice similarity coefficients were also significantly higher for super-resolved outputs than for up-sampled inputs across both structures (cerebral WM Dice: 0.911–0.922 vs. 0.896; cerebral cortex Dice: 0.848–0.867 vs. 0.771; average Dice: 0.879–0.894 vs. 0.834; two-sided *t*-test, $P < 0.001$; Fig. S4B). Increasing noise levels in the training references led to only a marginal reduction in segmentation performance. Dice similarity coefficients for all 12 individual brain structures and for all structures combined are reported in Table S3.

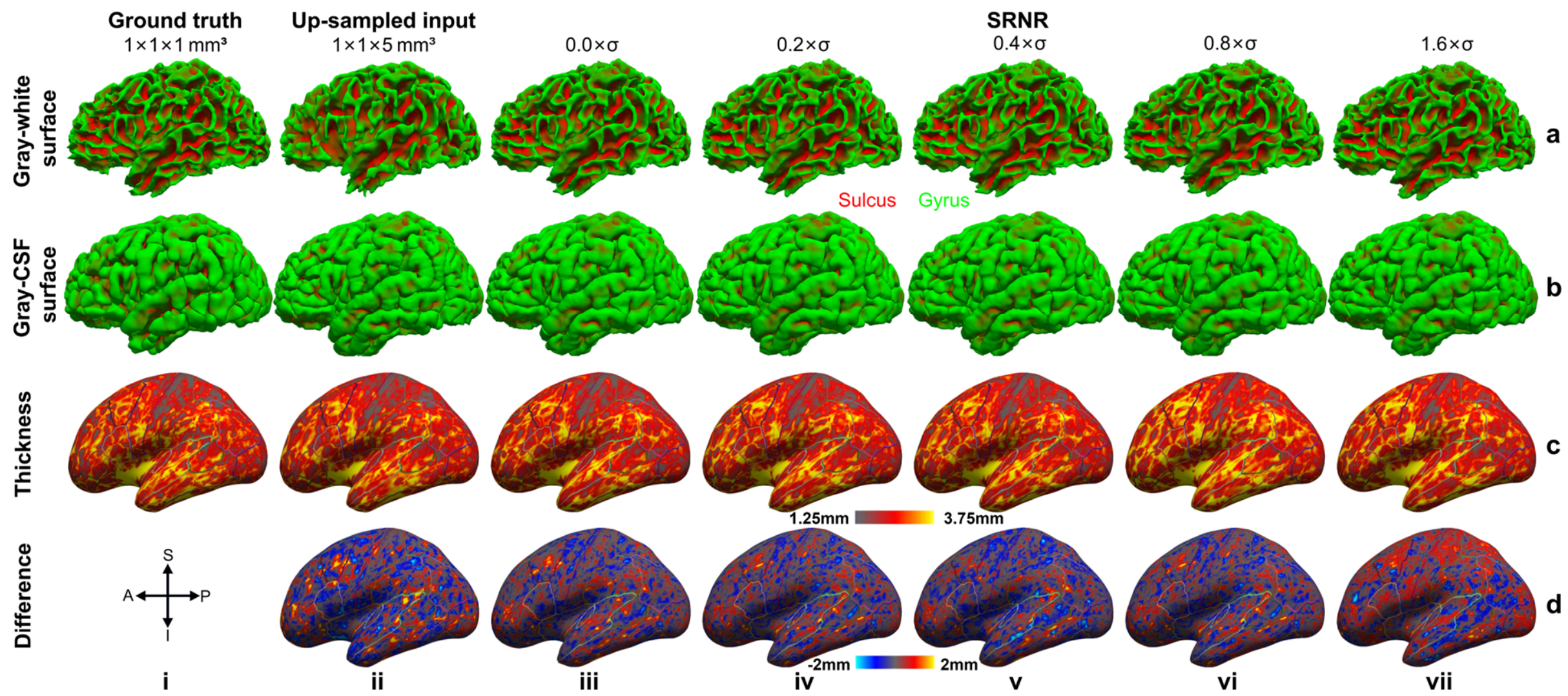

MAE (mm)	Up-sampled input 1×1×5 mm³	SRNR				
		**0.0×σ**	**0.2×σ**	**0.4×σ**	**0.8×σ**	**1.6×σ**
**Cortical thickness**	0.568 ± 0.063	0.450 ± 0.032	0.448 ± 0.055	0.456 ± 0.062	0.474 ± 0.068	0.475 ± 0.063
**Gray-white surface**	0.715 ± 0.103	0.592 ± 0.045	0.603 ± 0.089	0.618 ± 0.117	0.608 ± 0.124	0.638 ± 0.114
**Gray-CSF surface**	0.811 ± 0.237	0.497 ± 0.047	0.513 ± 0.042	0.532 ± 0.073	0.516 ± 0.029	0.548 ± 0.074

Fig. S5. Cortical surface reconstruction and cortical thickness accuracy in simulation experiments. Cortical surface reconstruction results are shown for a representative subject in the independent test set, including gray-white matter interface surfaces (a) and gray-CSF interface surfaces (b) reconstructed from the high-resolution, noise-free ground-truth image volume (i), the up-sampled thick-slice image volume (ii), and super-resolved outputs (iii-vii) from neural networks trained with reference data at varying noise levels. Left-hemispheric vertex-wise cortical thickness (c) and its difference relative to the ground truth (d) are displayed on the inflated surface. Different cortical regions defined by the FreeSurfer cortical parcellation (i.e., aparc.annot) are depicted as colored outlines. Group means (± standard deviations) of the whole-brain-averaged absolute difference in cortical thickness estimation and the displacements of the gray-white and gray-CSF surfaces estimated from the up-sampled and super-resolved outputs relative to the ground truth are quantified.

SRNR further improved cortical surface reconstruction accuracy, resulting in more accurate brain morphometric measurements (Fig. S5). Although cortical surfaces reconstructed from up-sampled low-resolution image volumes preserved the overall geometry of the ground truths, they exhibited distortions in fine structural details. In contrast, super-resolved outputs generated by SRNR showed improved surface continuity and clearer delineation of gyral and sulcal boundaries, yielding reconstructions

that were smoother and more consistent with the high-resolution references. These improvements were further corroborated by quantitative analyses. Compared with up-sampled image volumes, super-resolved outputs achieved lower errors in cortical thickness estimation (0.450–0.475 vs. 0.568), gray-white matter boundary positioning (0.592–0.638 vs. 0.715), and gray-CSF boundary positioning (0.497–0.548 vs. 0.811). Importantly, these gains were preserved across increasing noise levels in the training references, indicating robust morphometric performance of SRNR under noisy supervision.

Supplementary Note 2: Empirical experiment results using non-BM4D-denoised high-resolution as evaluation reference

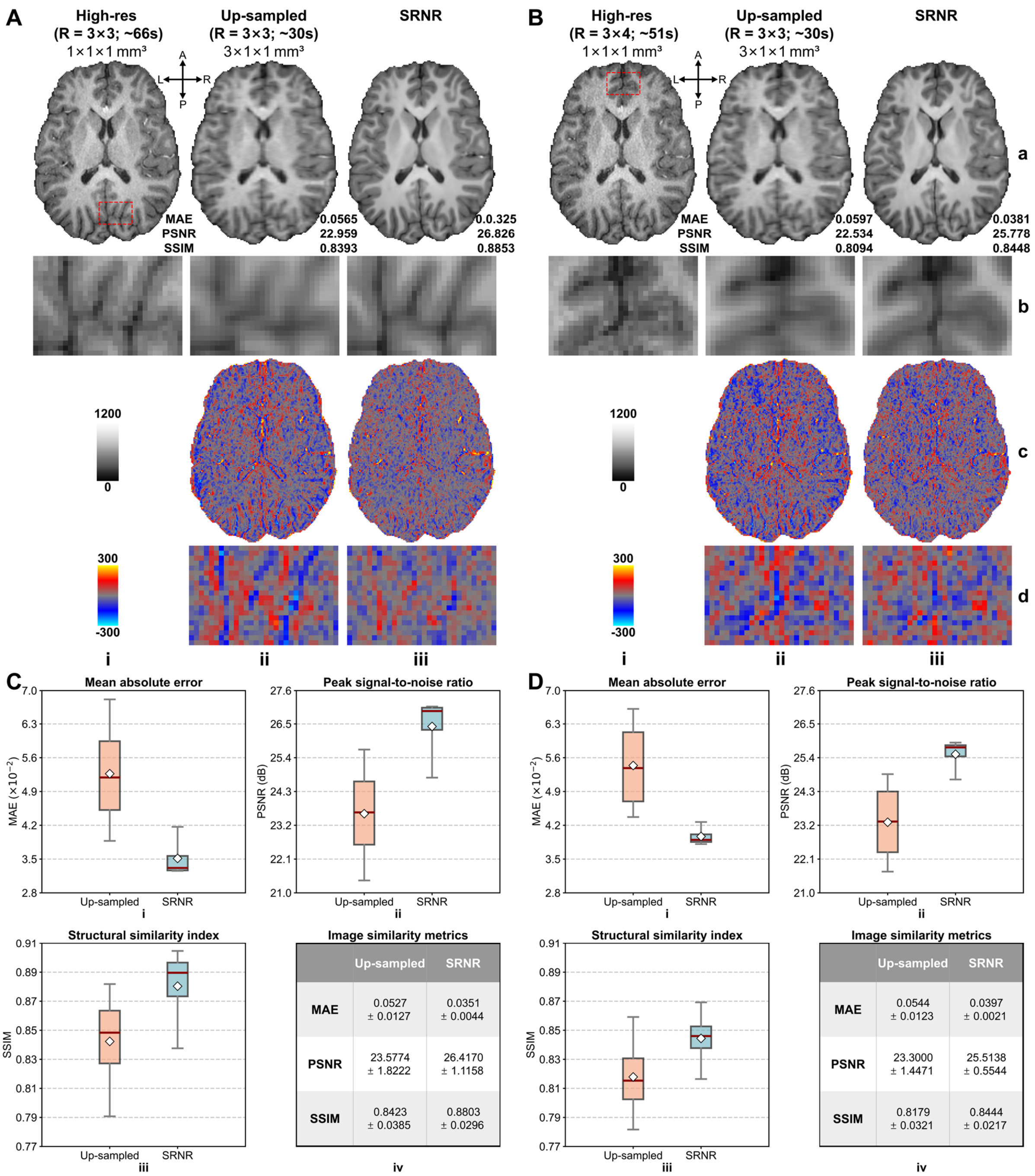

Fig. S6. Empirical experiments with data acquired at an acceleration factor of R = 3×3 (A, C) and R = 3×4 (B, D) using non-BM4D-denoised high-resolution as evaluation reference. A, B. Exemplar coronal slices from a representative pediatric clinical subject in the independent test set illustrate the acquired accelerated high-

resolution image volume (a, i), the up-sampled sagittal thick-slice image volume (a, ii), and the super-resolved output (a, iii), along with enlarged views (b). Difference maps depict the similarity of the up-sampled and super-resolved image volumes relative to the accelerated high-resolution image volume (c, d). Whole-brain-averaged MAE, PSNR, and SSIM for the exemplar subject are listed to quantitatively assess image similarity. **C, D.** Box plots illustrate the whole-brain-averaged MAE (i), PSNR (ii), and SSIM (iii) across four evaluation subjects, computed for up-sampled thick-slice image volumes (up-sampled) and super-resolved outputs, relative to the accelerated high-resolution reference image volumes. The midline indicates the median, diamond markers denote the mean values, box edges denote the first and third quartiles, and whiskers represent the minimum and maximum values. The corresponding quantitative values are listed (iv).

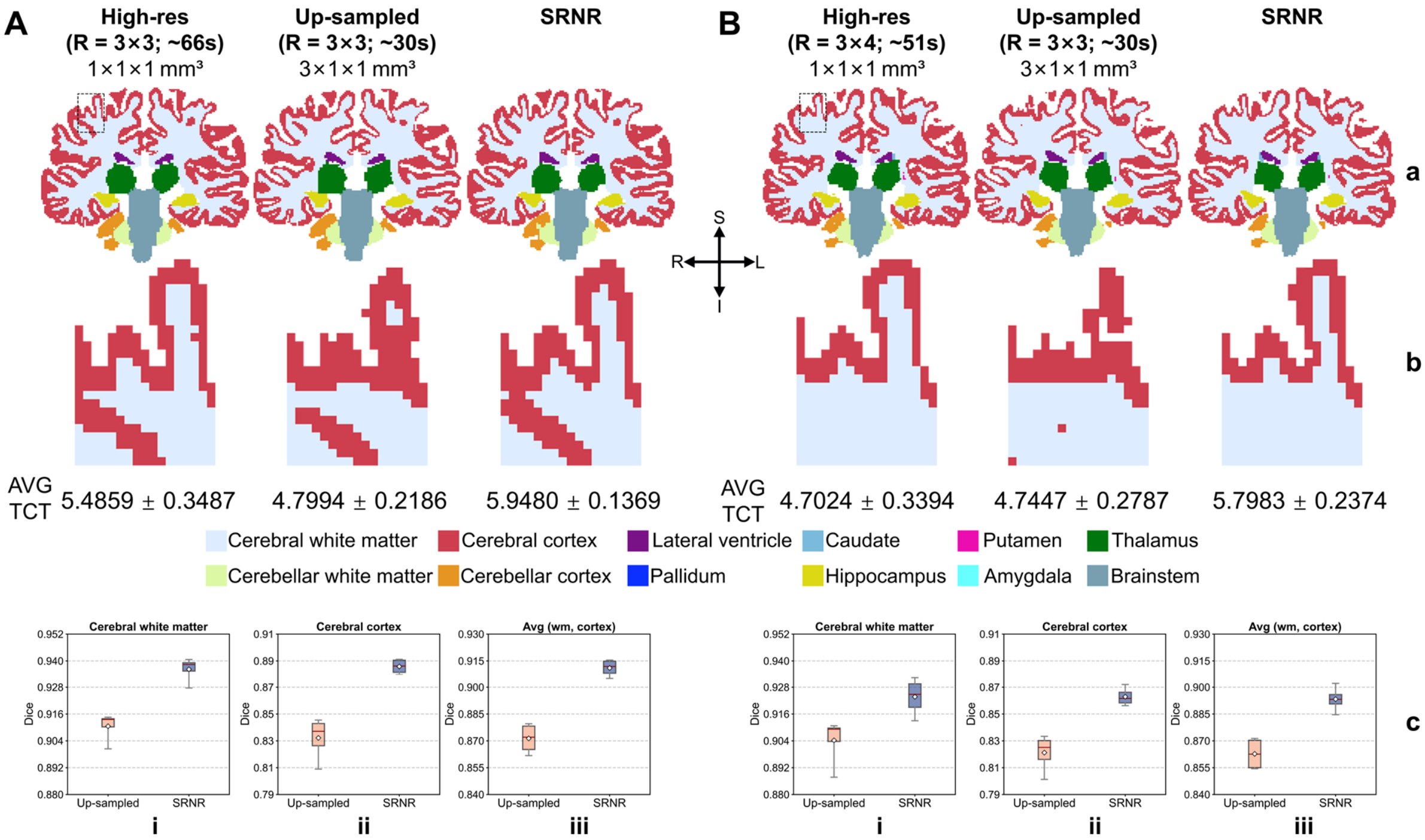

A:

	Up-sampled	SRNR
Cerebral white matter	0.9105 ± 0.0058	0.9363 ± 0.0051
Cerebral cortex	0.8322 ± 0.0143	0.8858 ± 0.0050
Average	0.8714 ± 0.0075	0.9111 ± 0.0043

B:

	Up-sampled	SRNR
Cerebral white matter	0.9042 ± 0.0095	0.9237 ± 0.0074
Cerebral cortex	0.8212 ± 0.0123	0.8629 ± 0.0060
Average	0.8627 ± 0.0079	0.8933 ± 0.0062

d

Fig. S7. Volumetric segmentation in empirical experiments with data acquired at an acceleration factor of R = 3×3 (A) and R = 3×4 (B) using non-BM4D-denoised high-resolution as evaluation reference. Exemplar coronal slices from a representative pediatric clinical subject show segmented brain structures derived from the high-SNR Wave-MPRAGE reference image volume (i), up-sampled thick-slice image volume (ii), and super-resolved output (iii), with enlarged views and average TCT values provided for comparison (b). Box plots illustrate the group mean (± standard deviation) Dice similarity coefficients across four evaluation subjects, computed for segmentations derived from up-sampled thick-slice image volumes and super-resolved outputs for the cerebral white matter (i), cerebral cortex (ii), and their average (iii). The midline indicates the median,

box edges indicate the first and third quartiles, and whiskers denote the minimum and the maximum values. Dice similarity coefficients for all 12 individual brain structures and for all structures combined are reported in Table S2.

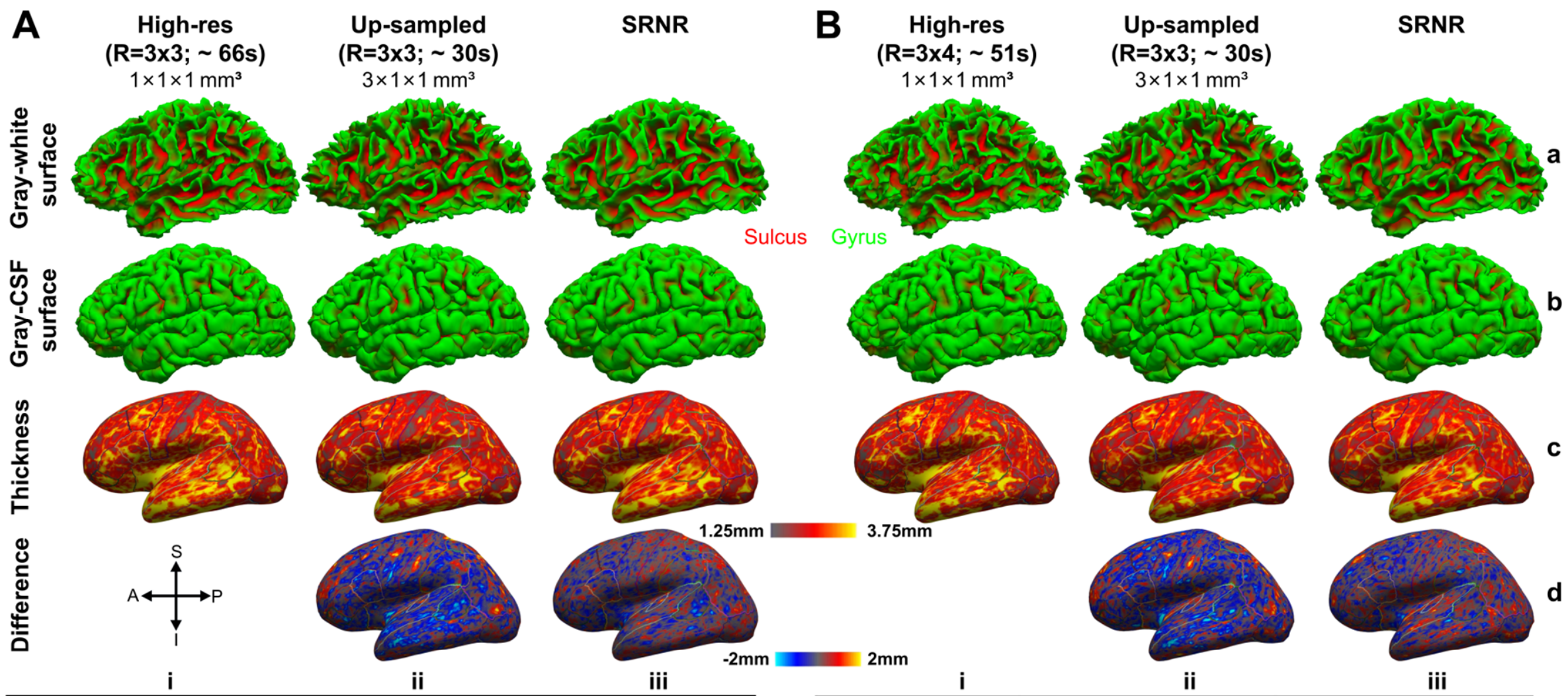

MAE (mm)	Up-sampled (R=3x3; ~ 30s) 3×1×1 mm³	SRNR
Cortical thickness	0.487 ± 0.021	0.384 ± 0.050
Gray-white surface	0.509 ± 0.019	0.415 ± 0.070
Gray-CSF surface	0.636 ± 0.044	0.591 ± 0.148

MAE (mm)	Up-sampled (R=3x3; ~ 30s) 3×1×1 mm³	SRNR
Cortical thickness	0.501±0.018	0.403 ± 0.020
Gray-white surface	0.540 ± 0.026	0.437 ± 0.026
Gray-CSF surface	0.671 ± 0.056	0.595 ± 0.061

e

Fig. S8. Cortical surface reconstruction and cortical thickness accuracy in empirical experiments with data acquired at an acceleration factor of R = 3×3 (A) and R = 3×4 (B) using non-BM4D-denoised high-resolution as evaluation reference. Cortical surface reconstruction results are shown for a representative pediatric subject in the empirical dataset, including the gray-white matter interface surfaces (a) and gray-CSF interface surfaces (b) reconstructed from the high-SNR Wave-MPRAGE reference image volume (i), up-sampled image volume (ii), and super-resolved output (iii). Left-hemispheric vertex-wise cortical thickness (c) and its difference relative to the high-resolution reference (d) are displayed on the inflated surface. Different cortical regions defined by the FreeSurfer cortical parcellation (i.e., aparc.annot) are depicted as colored outlines. Group means (± standard deviations) of the whole-brain-averaged absolute differences in the cortical thickness estimation and the displacements of the gray-white and gray-CSF surfaces estimated from the up-sampled and super-resolved predictions relative to the reference are quantified (e).

Supplementary Note 3: Transfer learning experiments using different pre-training datasets

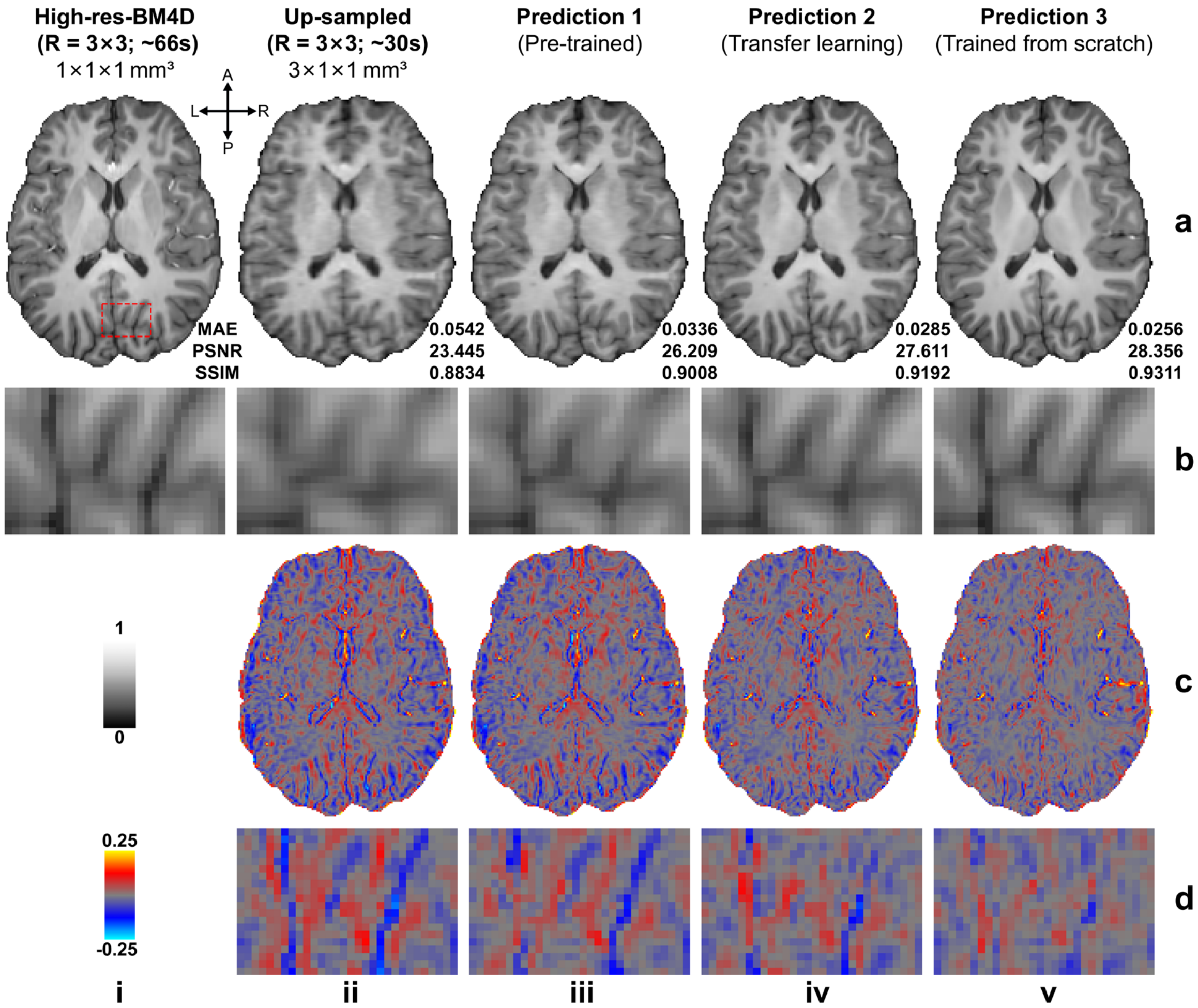

	Up-sampled	Pred 1	Pred 2	Pred 3
MAE	0.0493 ± 0.0137	0.0339 ± 0.0042	0.0299 ± 0.0035	0.0280 ± 0.0033
PSNR	24.2315 ± 2.0077	26.3137 ± 1.0949	27.4369 ± 1.1047	27.9555 ± 1.0868
SSIM	0.8864 ± 0.0283	0.9021 ± 0.0255	0.9170 ± 0.0220	0.9262 ± 0.0183

e

Fig. S9. Transfer learning results on empirical data acquired at an acceleration factor of R = 3×3 based on weights pre-trained on the HCP Dataset. Exemplar coronal slices from a representative pediatric clinical subject in the independent test set illustrate the acquired accelerated high-resolution reference image volume (a, i), the up-sampled sagittal thick-slice image volume (a, ii), the super-resolved prediction obtained by directly applying the pre-trained network (a, iii), the prediction after fine-tuning using three pediatric clinical cases (a, iv), and the prediction obtained by training from scratch using eight pediatric clinical cases (a, v). Enlarged views are provided for clarity (b). Difference maps depict the similarity of the up-sampled and super-resolved image volumes relative

to the accelerated high-resolution reference image volume (c, d). Whole-brain-averaged MAE, PSNR, and SSIM for the exemplar subject are listed to quantify image similarity. Box plots illustrate the whole-brain-averaged MAE (i), PSNR (ii), and SSIM (iii) across four evaluation subjects, computed for up-sampled thick-slice image volumes (up-sampled) and super-resolved outputs relative to the accelerated high-resolution reference image volumes (e). The midline indicates the median, diamond markers denote the mean values, box edges denote the first and third quartiles, and whiskers represent the minimum and maximum values. The corresponding quantitative values are listed (f).

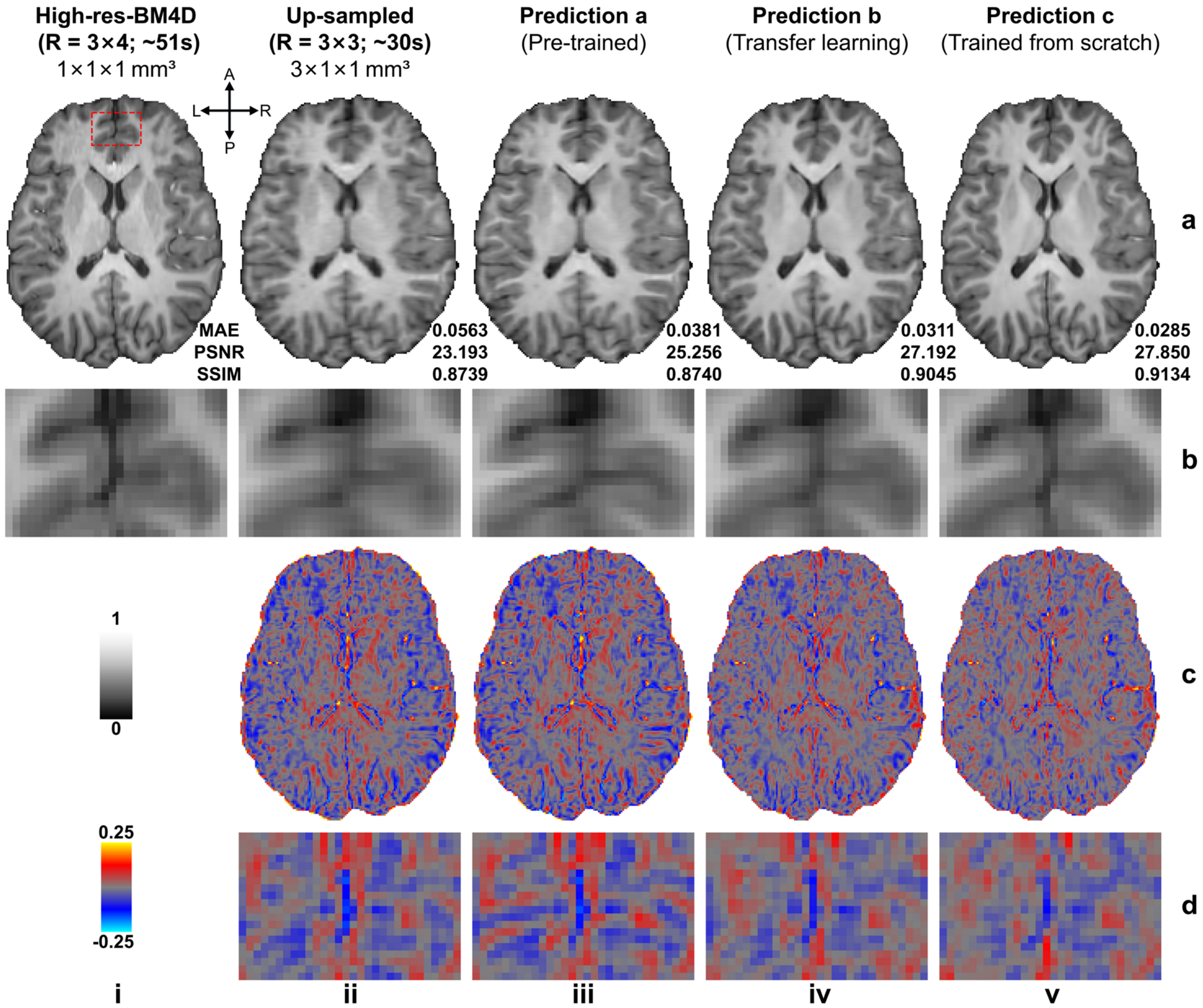

	Up-sampled	Pred a	Pred b	Pred c
MAE	0.0493 ± 0.0123	0.0377 ± 0.0024	0.0313 ± 0.0021	0.0299 ± 0.0017
PSNR	24.2487 ± 1.7812	25.4895 ± 0.7109	27.2406 ± 0.7684	27.5709 ± 0.6415
SSIM	0.8812 ± 0.0251	0.8795 ± 0.0214	0.9072 ± 0.0185	0.9111 ± 0.0145

e

Fig. S10. Transfer learning results on empirical data acquired at an acceleration factor of R = 3×4 based on weights pre-trained on the Calgary Preschool MRI Dataset. Exemplar coronal slices from a representative pediatric clinical subject in the independent test set illustrate the acquired accelerated high-resolution reference image

volume (a, i), the up-sampled sagittal thick-slice image volume (a, ii), the super-resolved prediction obtained by directly applying the pre-trained network (a, iii), the prediction after fine-tuning using three pediatric clinical cases (a, iv), and the prediction obtained by training from scratch using eight pediatric clinical cases (a, v). Enlarged views are provided for clarity (b). Difference maps depict the similarity of the up-sampled and super-resolved image volumes relative to the accelerated high-resolution reference image volume (c, d). Whole-brain-averaged MAE, PSNR, and SSIM for the exemplar subject are listed to quantify image similarity. Box plots illustrate the whole-brain-averaged MAE (i), PSNR (ii), and SSIM (iii) across four evaluation subjects, computed for up-sampled thick-slice image volumes (up-sampled) and super-resolved outputs relative to the accelerated high-resolution reference image volumes (e). The midline indicates the median, diamond markers denote the mean values, box edges denote the first and third quartiles, and whiskers represent the minimum and maximum values. The corresponding quantitative values are listed (f).

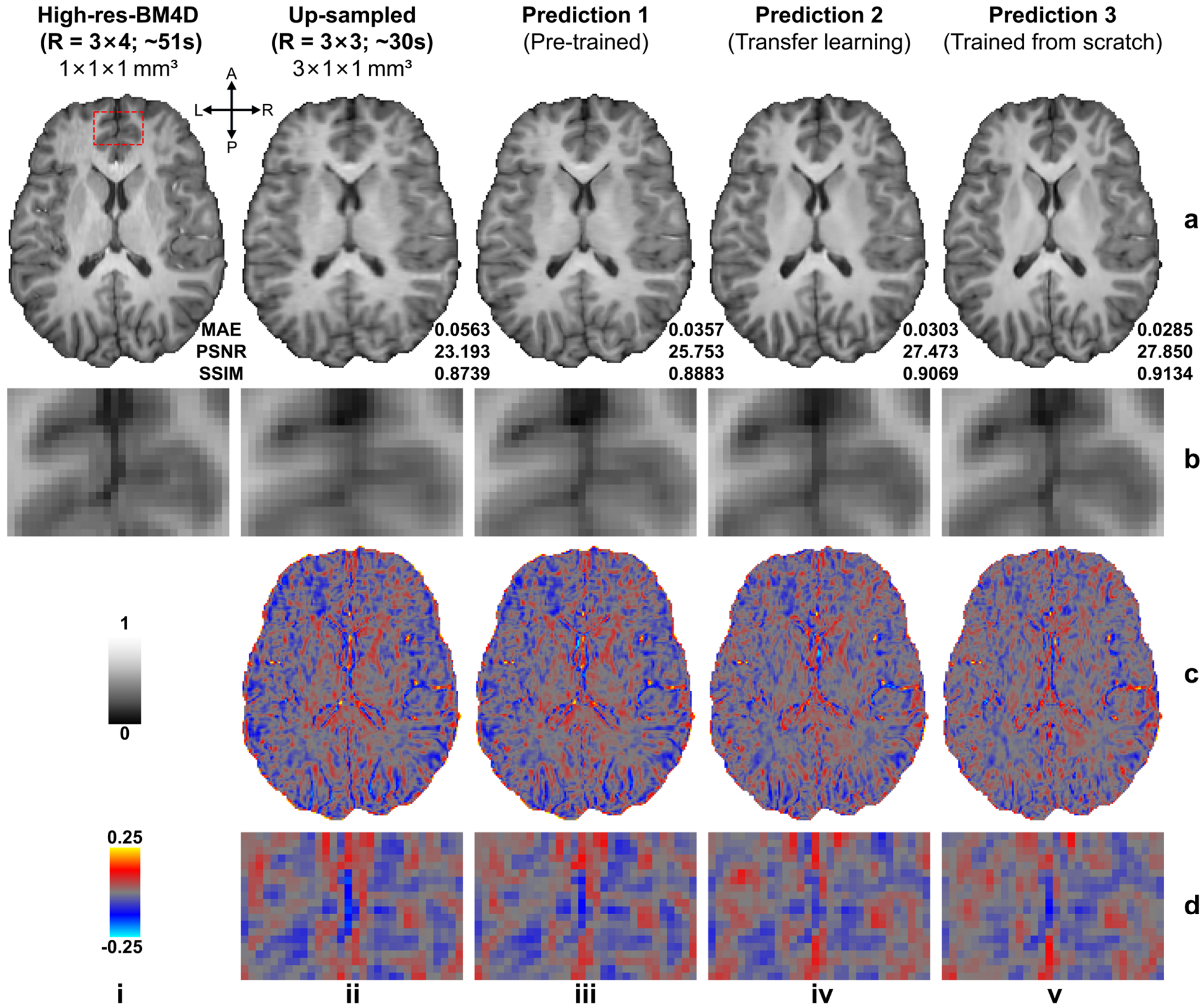

	Up-sampled	Pred a	Pred b	Pred c
**MAE**	0.0493 ± 0.0123	0.0350 ± 0.0027	0.0307 ± 0.0018	0.0299 ± 0.0017
**PSNR**	24.2487 ± 1.7812	26.0461 ± 0.7731	27.4385 ± 0.7161	27.5709 ± 0.6415
**SSIM**	0.8812 ± 0.0251	0.8942 ± 0.0214	0.9083 ± 0.0172	0.9111 ± 0.0145

e

Fig. S11. Transfer learning results on empirical data acquired at an acceleration factor of R = 3×4 based on weights pre-trained on the HCP Dataset. Exemplar coronal slices from a representative pediatric clinical subject in the independent test set illustrate the acquired accelerated high-resolution reference image volume (a, i), the up-sampled sagittal thick-slice image volume (a, ii), the super-resolved prediction obtained by directly applying the pre-trained network (a, iii), the prediction after fine-tuning using three pediatric clinical cases (a, iv), and the prediction obtained by training from scratch using eight pediatric clinical cases (a, v). Enlarged views are provided for clarity (b). Difference maps depict the similarity of the up-sampled and super-resolved image volumes relative to the accelerated high-resolution reference image volume (c, d). Whole-brain-averaged MAE, PSNR, and SSIM for the exemplar subject are listed to quantify image similarity. Box plots illustrate the whole-brain-averaged MAE (i), PSNR (ii), and SSIM (iii) across four evaluation subjects, computed for up-sampled thick-slice image volumes (up-

sampled) and super-resolved outputs relative to the accelerated high-resolution reference image volumes (e). The midline indicates the median, diamond markers denote the mean values, box edges denote the first and third quartiles, and whiskers represent the minimum and maximum values. The corresponding quantitative values are listed (f).

Supplementary Tables

Table S1. Dice similarity coefficients for all 12 individual brain structures and for all structures combined in simulation experiments using the publicly available Calgary Preschool MRI Dataset, computed from up-sampled low-resolution image volumes (ups) and super-resolved predictions from neural networks trained on high-resolution reference data with varying noise levels (0.0–1.6 × σ).

	Cerebral white mat	Cerebral cortex	Lateral ventricle	Cerebellar white mat	Cerebellar cortex	Thalamus	Caudate	Putamen	Pallidum	Hippo-campus	Amygdala	Brainstem	All
Ups	0.8073 ± 0.0606	0.7697 ± 0.0830	0.7898 ± 0.0258	0.8485 ± 0.0250	0.9165 ± 0.0109	0.9086 ± 0.0162	0.8661 ± 0.0345	0.9023 ± 0.0190	0.8860 ± 0.0222	0.8550 ± 0.0149	0.8619 ± 0.0241	0.9317 ± 0.0089	0.9382 ± 0.0302
0.0	0.9602 ± 0.0035	0.9468 ± 0.0040	0.9230 ± 0.0193	0.9543 ± 0.0052	0.9769 ± 0.0026	0.9534 ± 0.0081	0.9479 ± 0.0081	0.9619 ± 0.0040	0.9346 ± 0.0111	0.9460 ± 0.0065	0.9432 ± 0.0095	0.9709 ± 0.0058	0.9795 ± 0.0013
0.2	0.9597 ± 0.0034	0.9462 ± 0.0038	0.9253 ± 0.0191	0.9543 ± 0.0052	0.9766 ± 0.0024	0.9544 ± 0.0089	0.9492 ± 0.0090	0.9628 ± 0.0049	0.9381 ± 0.0121	0.9455 ± 0.0065	0.9433 ± 0.0075	0.9718 ± 0.0047	0.9794 ± 0.0013
0.4	0.9589 ± 0.0034	0.9446 ± 0.0044	0.9268 ± 0.0174	0.9540 ± 0.0048	0.9767 ± 0.0023	0.9557 ± 0.0084	0.9492 ± 0.0080	0.9622 ± 0.0037	0.9365 ± 0.0108	0.9454 ± 0.0069	0.9429 ± 0.0075	0.9724 ± 0.0055	0.9788 ± 0.0016
0.8	0.9540 ± 0.0034	0.9392 ± 0.0045	0.9244 ± 0.0157	0.9476 ± 0.0056	0.9742 ± 0.0025	0.9518 ± 0.0101	0.9479 ± 0.0072	0.9590 ± 0.0040	0.9321 ± 0.0097	0.9415 ± 0.0062	0.9402 ± 0.0078	0.9712 ± 0.0052	0.9771 ± 0.0016
1.6	0.9507 ± 0.0034	0.9354 ± 0.0040	0.9210 ± 0.0176	0.9442 ± 0.0055	0.9727 ± 0.0023	0.9488 ± 0.0094	0.9448 ± 0.0084	0.9581 ± 0.0042	0.9286 ± 0.0132	0.9392 ± 0.0060	0.9354 ± 0.0079	0.9703 ± 0.0041	0.9760 ± 0.0013

Table S2. Dice similarity coefficients for all 12 individual brain structures and for all structures combined in empirical experiments (R = 3×3 and R = 3×4). Metrics are reported using high-resolution image volumes after BM4D denoising as references, as well as using the original high-resolution image volumes as references.

3×3		Cerebral white mat	Cerebral cortex	Lateral ventricle	Cerebellar white mat	Cerebellar cortex	Thalamus	Caudate	Putamen	Pallidum	Hippo-campus	Amygdala	Brainstem	All
High-res	Ups	0.9105 ± 0.0058	0.8322 ± 0.0143	0.9168 ± 0.0212	0.8853 ± 0.0132	0.9250 ± 0.0109	0.9077 ± 0.0149	0.8952 ± 0.0207	0.8745 ± 0.0207	0.8279 ± 0.0246	0.8818 ± 0.0052	0.8860 ± 0.0076	0.9481 ± 0.0079	0.9506 ± 0.0058
	Pred	0.9363 ± 0.0051	0.8858 ± 0.0050	0.9409 ± 0.0162	0.8815 ± 0.0144	0.9316 ± 0.0082	0.9296 ± 0.0160	0.9210 ± 0.0141	0.9164 ± 0.0094	0.8664 ± 0.0245	0.8975 ± 0.0123	0.8904 ± 0.0067	0.9565 ± 0.0052	0.9656 ± 0.0026
High-res -BM4D	Ups	0.9130 ± 0.0058	0.8359 ± 0.0124	0.9183 ± 0.0199	0.8858 ± 0.0175	0.9284 ± 0.0154	0.9090 ± 0.0139	0.8957 ± 0.0198	0.8694 ± 0.0230	0.8297 ± 0.0179	0.8848 ± 0.0072	0.8926 ± 0.0120	0.9460 ± 0.0054	0.9532 ± 0.0049
	Pred	0.9389 ± 0.0046	0.8888 ± 0.0056	0.9393 ± 0.0196	0.8937 ± 0.0210	0.9331 ± 0.0083	0.9270 ± 0.0176	0.9225 ± 0.0153	0.9121 ± 0.0124	0.8674 ± 0.0178	0.8996 ± 0.0070	0.8915 ± 0.0145	0.9582 ± 0.0086	0.9667 ± 0.0029

3×4		Cerebral white mat	Cerebral cortex	Lateral ventricle	Cerebellar white mat	Cerebellar cortex	Thalamus	Caudate	Putamen	Pallidum	Hippo-campus	Amygdala	Brainstem	All
High-res	Ups	0.9042 ± 0.0095	0.8212 ± 0.0123	0.9190 ± 0.0194	0.8698 ± 0.0101	0.9277 ± 0.0078	0.9172 ± 0.0126	0.8938 ± 0.0187	0.8538 ± 0.0293	0.8087 ± 0.0328	0.8751 ± 0.0172	0.8671 ± 0.0047	0.9437 ± 0.0106	0.9481 ± 0.0044
	Pred	0.9237 ± 0.0074	0.8629 ± 0.0060	0.9359 ± 0.0201	0.8826 ± 0.0093	0.9314 ± 0.0089	0.9244 ± 0.0127	0.9143 ± 0.0075	0.8948 ± 0.0084	0.8372 ± 0.0217	0.8837 ± 0.0071	0.8707 ± 0.0074	0.9503 ± 0.0080	0.9591 ± 0.0023
High-res -BM4D	Ups	0.9088 ± 0.0069	0.8275 ± 0.0170	0.9192 ± 0.0195	0.8749 ± 0.0122	0.9278 ± 0.0122	0.9080 ± 0.0190	0.8884 ± 0.0250	0.8558 ± 0.0274	0.8140 ± 0.0264	0.8713 ± 0.0132	0.8704 ± 0.0041	0.9428 ± 0.0112	0.9511 ± 0.0055
	Pred	0.9305 ± 0.0057	0.8728 ± 0.0089	0.9398 ± 0.0188	0.8908 ± 0.0117	0.9351 ± 0.0085	0.9222 ± 0.0126	0.9152 ± 0.0116	0.9000 ± 0.0099	0.8391 ± 0.0210	0.8842 ± 0.0057	0.8728 ± 0.0084	0.9519 ± 0.0082	0.9630 ± 0.0034

Table S3. Dice similarity coefficients for all 12 individual brain structures and for all structures combined in simulation experiments using the publicly available HCP dataset, computed from up-sampled low-resolution image volumes (ups) and super-resolved predictions from neural networks trained on high-resolution reference data with varying noise levels (0.0–1.6 × σ).

	Cerebral white mat	Cerebral cortex	Lateral ventricle	Cerebellar white mat	Cerebellar cortex	Thalamus	Caudate	Putamen	Pallidum	Hippo-campus	Amygdala	Brainstem	All
Ups	0.8960 ± 0.0067	0.7712 ± 0.0097	0.8516 ± 0.0400	0.8408 ± 0.0128	0.9015 ± 0.0113	0.8827 ± 0.0207	0.8639 ± 0.0222	0.8894 ± 0.0239	0.8345 ± 0.0336	0.7617 ± 0.0391	0.8263 ± 0.0325	0.9221 ± 0.0098	0.9324 ± 0.0048
0.0×σ	0.9215 ± 0.0119	0.8670 ± 0.0188	0.9368 ± 0.0216	0.8161 ± 0.0354	0.9116 ± 0.0225	0.8955 ± 0.0211	0.8687 ± 0.0436	0.8059 ± 0.0579	0.7485 ± 0.0793	0.8419 ± 0.0320	0.8197 ± 0.0559	0.9307 ± 0.0179	0.9684 ± 0.0017
0.2×σ	0.9194 ± 0.0097	0.8613 ± 0.0165	0.9344 ± 0.0225	0.8129 ± 0.0318	0.9095 ± 0.0199	0.8939 ± 0.0176	0.8700 ± 0.0429	0.8112 ± 0.0539	0.7522 ± 0.0886	0.8386 ± 0.0265	0.8262 ± 0.0477	0.9339 ± 0.0104	0.9667 ± 0.0018
0.4×σ	0.9193 ± 0.0085	0.8612 ± 0.0152	0.9349 ± 0.0213	0.8069 ± 0.0270	0.9073 ± 0.0169	0.8938 ± 0.0173	0.8743 ± 0.0381	0.8121 ± 0.0522	0.7440 ± 0.0855	0.8378 ± 0.0291	0.8162 ± 0.0525	0.9306 ± 0.0127	0.9662 ± 0.0018
0.8×σ	0.9189 ± 0.0114	0.8587 ± 0.0190	0.9351 ± 0.0215	0.8125 ± 0.0292	0.9091 ± 0.0206	0.8901 ± 0.0172	0.8726 ± 0.0415	0.8209 ± 0.0580	0.7552 ± 0.0742	0.8424 ± 0.0286	0.8234 ± 0.0529	0.9330 ± 0.0117	0.9656 ± 0.0018
1.6×σ	0.9105 ± 0.0102	0.8475 ± 0.0178	0.9338 ± 0.0244	0.7975 ± 0.0254	0.9018 ± 0.0166	0.8873 ± 0.0137	0.8572 ± 0.0439	0.8086 ± 0.0475	0.7463 ± 0.0648	0.8219 ± 0.0299	0.8132 ± 0.0478	0.9269 ± 0.0120	0.9653 ± 0.0019

References

Cocosco, C.A., Kollokian, V., Kwan, R.K.-S., & Evans, A.C. (1997). BrainWeb: Online Interface to a 3D MRI Simulated Brain Database. *NeuroImage, 5(4), S425.* https://cir.nii.ac.jp/crid/1370846644342763397

Fischl, B. (2012). FreeSurfer. *NeuroImage*, *62*(2), 774–781. https://doi.org/10.1016/j.neuroimage.2012.01.021

Glasser, M. F., Smith, S. M., Marcus, D. S., Andersson, J. L. R., Auerbach, E. J., Behrens, T. E. J., Coalson, T. S., Harms, M. P., Jenkinson, M., Moeller, S., Robinson, E. C., Sotiropoulos, S. N., Xu, J., Yacoub, E., Ugurbil, K., & Van Essen, D. C. (2016). The Human Connectome Project's neuroimaging approach. *Nature Neuroscience*, *19*(9), 1175–1187. https://doi.org/10.1038/nn.4361

Glasser, M. F., Sotiropoulos, S. N., Wilson, J. A., Coalson, T. S., Fischl, B., Andersson, J. L., Xu, J., Jbabdi, S., Webster, M., Polimeni, J. R., Van Essen, D. C., & Jenkinson, M. (2013). The minimal preprocessing pipelines for the Human Connectome Project. *NeuroImage*, *80*, 105–124. https://doi.org/10.1016/j.neuroimage.2013.04.127

Kingma, D. P., & Ba, J. (2017). *Adam: A Method for Stochastic Optimization* (arXiv:1412.6980). arXiv. https://doi.org/10.48550/arXiv.1412.6980

Postelnicu, G., Zollei, L., & Fischl, B. (2009). Combined Volumetric and Surface Registration. *IEEE Transactions on Medical Imaging*, *28*(4), 508–522. https://doi.org/10.1109/TMI.2008.2004426
